# Components of Total Energy Expenditure in Healthy and Critically Ill Children: A Comprehensive Review

**DOI:** 10.3390/nu16162581

**Published:** 2024-08-06

**Authors:** Georgia A. Parshuram, Lori Tuira, Frances Dazo, Noura El Hariri, Jessie M. Hulst, Haifa Mtaweh

**Affiliations:** 1 Faculty of Medicine, Dentistry, and Health Sciences, University of Melbourne, Melbourne, VIC 3052, Australia; 2 Department of Clinical Dietetics, The Hospital for Sick Children, Toronto, ON M5G 1X8, Canada; 3 Department of Critical Care, The Hospital for Sick Children, Toronto, ON M5G 1X8, Canada; 4 Division of Gastroenterology, Hepatology, and Nutrition, The Hospital for Sick Children, Toronto, ON M5G 1X8, Canada; 5 Department of Pediatrics, University of Toronto, Toronto, ON M5S 1A8, Canada; 6 Department of Nutritional Sciences, University of Toronto, Toronto, ON M5S 1A8, Canada

**Keywords:** total energy expenditure, children, activity energy expenditure, growth energy expenditure, thermic effect of feeding

## Abstract

Background: Total energy expenditure (TEE) is the total energy expended by an individual to sustain life, activities, and growth. TEE is formed by four components: resting energy expenditure (REE), activity energy expenditure (AEE), growth-related energy expenditure (GEE), and the thermic effect of feeding (TEF). Some energy expenditure (EE) components may change throughout childhood and cannot be reliably estimated using prediction formulae. Objective: To summarize measured TEE components as reported in the literature in healthy and critically ill children. Methods: We searched MEDLINE, EMBASE, and CINAHL for studies published between 1946 and 7 September 2023. The primary outcome was energy expenditure. Included studies were published in English and measured one or more of TEE, AEE, GEE, and TEF with Indirect Calorimetry or Doubly Labeled Water in participants between 1 month and 18 years of age. We excluded studies reporting only REE or using predictive equations. Following abstraction, reported values were converted into kcal/kg/day or kcal/day as possible. Weighted mean values were calculated using median or means of EE measurements. Results: We found 138 studies, 8163 patients, and 16,636 eligible measurements. The median (IQR) study sample size was 20 (12, 35) patients. TEE was the most evaluated component. The median (IQR) TEE in infants was 73.1 (67.0, 76.5), in children 78.0 (66.0, 81.3), and in adolescents was 44.2 (41.8, 51.9) kcal/kg/day. Very few studies reported on GEE and TEF. Conclusions: This is one of the first studies that summarizes components of total energy expenditure in different pediatric age groups in healthy and critically ill children. Growth- and feeding-associated energy expenditure are poorly reported in healthy children, while all components of TEE (except REE) are poorly reported in critically ill children.

## 1. Introduction

Energy is expended in childhood to support basal metabolism, physical activity, growth, and the thermic effect of feeding. These can be measured as resting energy expenditure (REE), activity-related energy expenditure (AEE), growth-related energy expenditure (GEE), and the thermic effect of feeding (TEF). Together these components form total energy expenditure (TEE) [1]. TEE changes throughout childhood and reflects differences in body size and composition, physical activity, and growth. Growth is initially rapid, slows in middle childhood, and then accelerates in adolescence [2,3]. Thermic effect of feeding similarly decreases beyond childhood years and is dependent on the size of both the meal and the adiposity of the individual [4]. Activity and basal metabolism may vary depending on age, sex, and health status [5,6,7]. 

The contribution of each component to TEE Is variable. REE is reported to account for 60–70% of TEE, AEE for 30–40% of TEE depending on level of physical activity, and TEF for around 10% of TEE [4,8]. GEE is comprised of “Esynthesis”, the energy required to produce new tissues and “Edeposited”, the energy deposited in the new formed tissues [9]. GEE is the most variable component of TEE, accounting for up to 60% of TEE in the first month of life, decreasing to around 1–2% of TEE at 2 years of age, increasing slightly prepubertally and then again during puberty, whereafter it drops to 0% in mid-twenties when individuals generally stop growing [2,10]. 

Methods to measure components of energy expenditure include Doubly Labeled Water (DLW) and Direct and Indirect Calorimetry [11]. Calorimetry utilizes the measurement of oxygen consumption and carbon dioxide production over a period of 30 min to an hour to calculate energy expenditure [12]. DLW is currently the reference standard to determine TEE. It utilizes labelled oxygen and hydrogen atoms to determine oxygen consumption and carbon dioxide production, and it requires 1–2 weeks to allow turnover of these atoms [12]. All require specialized equipment and have limited use in the daily care of hospitalized children. Accordingly, prediction equations derived from healthy children are often used with correction factors for those hospitalized with different disease pathologies. These equations have been shown to be imprecise in acute illness [13,14]. Children hospitalized with critical illness go through different phases of metabolism, where initial stages could be adequately represented by REE. As time passes by, energy expenditure directed for recovery and potentially growth can be relevant for the care of critically ill children [15]. Interventions without an accurate understanding of energy expenditure can lead to effects of worsening under-nutrition and over-nutrition with their associated morbidities such as malnutrition, infection, and growth failure, or conversely, fatty liver and obesity [16,17]. REE in health and disease, its variability across different age groups, and factors associated with changes in REE have been summarized in the literature [18,19,20,21]; however, less data are available on the contribution of other components to TEE. In this report, we summarize the available data of the objectively measured components of total energy expenditure in term neonates, infants, children, and adolescents in states of health and critical illness.

## 2. Materials and Methods

We conducted a literature review of articles published between 1946 and 7 September 2023. We searched the following databases: MEDLINE, EMBASE, and CINAHL. These were complemented by a reference review of articles included after the preliminary search. Initial keywords included energy expenditure, doubly labeled water, and indirect calorimetry. The full set of search terms is available in the Appendix A (Table A1). 

Eligible studies were included if they were peer-reviewed, published in an English language journal, and reported on one or more of TEE, AEE, GEE, and TEF. Eligible participants were children from 1 month to 18 years of age. Eligible studies reported measurements of TEE and its components with Indirect Calorimetry or Doubly Labeled Water. We included studies where AEE was calculated after direct measurement of other components of TEE. We excluded studies that only measured REE or basal metabolic rate, that only used predictive equations to calculate energy expenditure, and that studied elite athletes, preterm neonates, or participants with chronic disease. Narrative reviews, editorials, letters, commentaries, guidelines, or grey literature were excluded. References were imported in EndNote20 (Clarivate), duplicates were identified and removed, then title and abstract screening identified articles for full text screening. 

Data were abstracted from full texts and managed as follows. Study design was classified as interventional or observational, prospective or retrospective, and cross-sectional to represent studies evaluating patients at one time point or longitudinal where patients underwent multiple time-point evaluations. Additional data included number of participants and patient age at time of enrollment, and were categorized into the following categories: neonate (0–30 days), infant (1 month–1 year), child (1 year–10 years), adolescent (>10 years), sex (male/female/mixed), disease and health state (healthy, healthy overweight/obese, healthy with burns), and critically ill (critically ill); if a study included both healthy and healthy obese, it was classified as healthy obese in the descriptive components of this review. The above categorization of health and disease states was based on the literature review that suggests that healthy obese patients are physiologically different in regards to energy expenditure from non-obese patients, the healthy with burns category is based on the classical description of these patients as hypermetabolic, and critically ill patients were a specific area of interest for our group, but the number of studies was small to prevent a more detailed description of the critical illness. The maximum enrolled number of patients was used to represent the sample size summary of the overall studies. Groups of patients were defined based on the following hierarchical order of variables: sex, then age category of participants. We abstracted the measured energy expenditure component, method of measurement, conditions during which EE was measured (meal and activity type), and the reported unit (kcal/kg/day was the preferred unit, but if unavailable, we abstracted the unit as reported then converted the unit to kcal/kg/day or kcal/day). In studies reporting on AEE, we classified the activity as either high-intensity or low-intensity activity based on the authors description of the activity. Low-intensity activities included slow-pace walking or playing with a toy, while examples of high intensity included playing catch or brisk walking. Measures of central tendency and spread were abstracted from the studies for study groups and TEE and its components. We identified the average values of EE measurements and ages using a hierarchical approach that preferred the mean over median values and the mid-point of any reported range if neither the mean or median was available. The weighted mean and its standard error were calculated from the mean and sample size of studies. The data were summarized by age groups, sex, EE component measured, and disease state. 

## 3. Results

**Figure 1:**
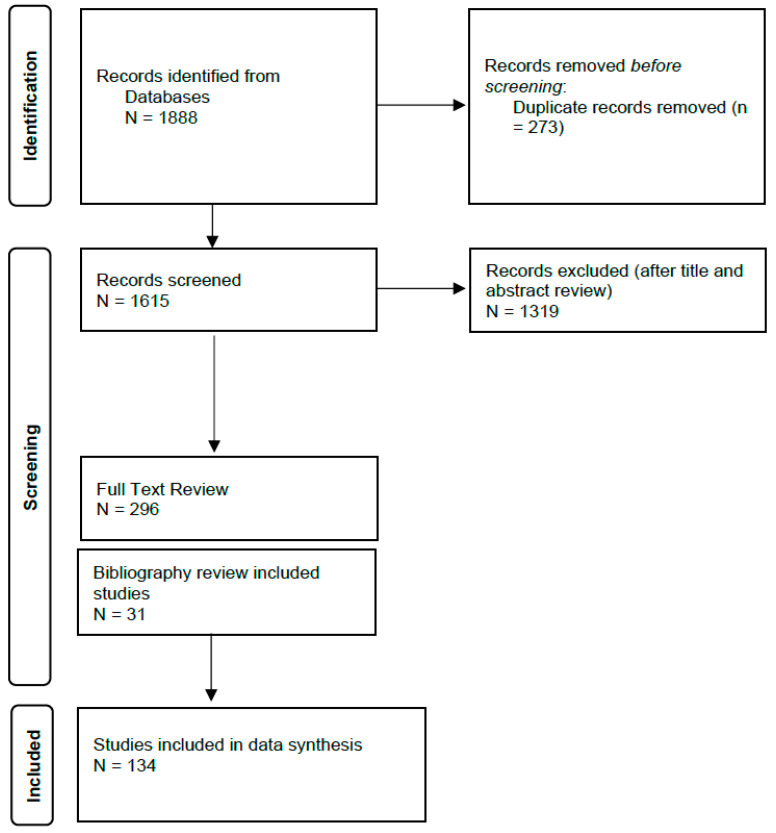
Flowchart for study selection.

The search strategy yielded 1615 unique articles; 296 underwent full text review. The bibliography search provided 31 additional eligible studies, and 134 studies were included for data synthesis (Figure 1). A summary of the included studies is presented in Table S1. 

Of the 134 included studies, 130 studies (97%) evaluated healthy children (obese, overweight, burns post-recovery, stunted, short stature) and 4 studies (3%) evaluated critically ill children. The study design was prospective observational in all included studies, cross-sectional in 127 (95%) or longitudinal in 7 (5%) studies. A total of 8163 patients were included with 16,636 eligible measurements. Measurement techniques were doubly labelled water in 71 (53%) studies, indirect calorimetry in 15 (10%) studies, and both doubly labelled water and indirect calorimetry in 48 (36%) studies (Table S1). The median (IQR) age across all studies was 114 (63.6, 156) months and the median (IQR) sample size was 20 (12, 35) patients (Table 1). Our search did not identify any studies conducted only in neonates or that measured any of the TEE components of interest.

**Figure 2:**
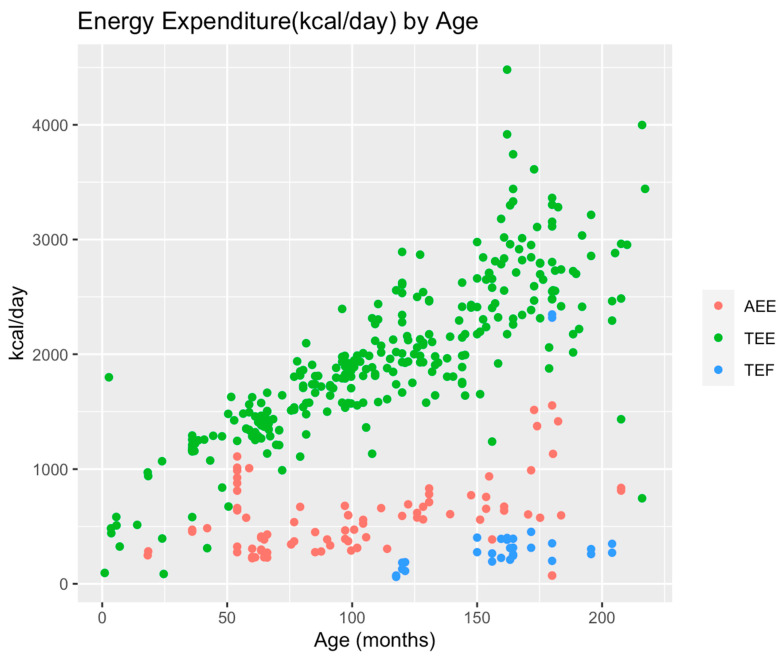
Energy Expenditure (kcal/day) by Age. Total energy expenditure increased with increasing patient age, while activity energy expenditure and thermic effect of feeding were not correlated to age. TEE: Total energy expenditure, AEE: Activity-related energy expenditure, TEF: thermic effect of feeding.

Activity energy expenditure (AEE) was evaluated in 42 studies, with 4631 measurements in 2706 subjects. Most of the measurements were performed in the child and adolescent age categories (N = 3754, 81%), and all measurements were conducted in conditions of health. The median sample size across studies was 15 (11, 28) subjects. Amongst the measurements performed in kcal/kg/day, the median (Q1, Q3) AEE from 78 measurements in infants was 15.0 (13.3, 17.7), from 73 measurements in children was 18.5 (17.3, 20.2), and from 90 measurements in adolescents was 41.3 (18.3, 77.1). Amongst the AEE measurements reported in kcal/day, median (Q1, Q3) AEE from 1711 measurements in children was 1010.4 (403.5, 2880), and from 1880 measurements in adolescents was 2404 (761.3, 4140) (Table 2). Low-intensity activity results in 15–24 kcal/kg/day of energy expenditure. Thermic effect of feeding-related energy expenditure (TEF) was evaluated in four studies, 59 subjects, with 80 measurements. Studies were performed in healthy adolescents or mixed-age categories that could not be separated. The median (Q1, Q3) TEF-EE from 60 measurements in adolescents was 289.5 (222, 386) kcal/day. Growth-associated energy expenditure (GEE) was measured in one study that included 12 infants; with 24 measurements, the median (Q1, Q3) GEE was 26.3 (25.1, 27.5) kcal/kg/day. Total energy expenditure (TEE) was measured in 7652 subjects from 124 studies, with a total of 7750 measurements. These were predominantly in healthy children and reported in kcal/day. In those where the measurement was reported in kcal/kg/day, the median (Q1, Q3) TEE for infants was 73.1 (67, 76.5) kcal/kg/day, for the child age category was 78 (66, 81.3) kcal/kg/day, and for adolescents, TEE was 44.2 (41.8, 51.9) kcal/kg/day (Table 2, Figure 2). 

## 4. Discussion

This review is one of the first published reviews summarizing the available literature on the measurement of total energy expenditure components in both healthy and critically ill children. We highlight three main findings. 

First, we have found a predominance of studies reporting on total energy expenditure, followed by studies reporting on activity-associated energy expenditure. There is a scarcity of data reported on growth-associated EE and thermic effect of feeding. Studies of these components of total energy expenditure are predominantly in healthy children, with 5 of 134 (4%) studies being performed in critically ill children, and all measuring total energy expenditure. 

Second, TEE and age demonstrate a linear relationship with an increase in TEE during infancy and childhood, then a reduction in adolescence when TEE is standardized to weight in kg. This could be explained by the energy directed towards growth and somatic tissue deposition that is of a higher rate during early years with an eventual reduction in later years. This energy is predominantly driven by fat-free mass deposition. This is in keeping with what has been reported in the literature [22,23]. 

Third, activity energy expenditure could contribute significantly to total energy expenditure even if the activity is classified as low intensity. The contribution of this component, particularly if patients remain active as they develop acute illness or in rehabilitation after critical illness, needs to be adequately considered if not evaluated. 

The limitations of this review include some that are modifiable and some that are limited by the nature of the underlying studies available for review. First, the limited number of studies that evaluated GEE and TEF prevent the data available on these two components from being useful in the clinical context. Second, we utilized the authors categorization as healthy and critically ill children. Therefore, this categorization is liable to the bias of where the study is performed and how the clinicians have classified the patient population. Third, we did not explore the difference between doubly labelled water and indirect calorimetry measurements of total energy expenditure and its components; this might be of added value to define the preferred method for determination of each of those components. Fourth, the nutritional state of children evaluated in these studies was not extracted and the interpretation of TEE results needs to account of the lack of those data. Finally, there is a large number of studies of small sample size, with wide age ranges spanning different patient age groups, and different methodologies for energy expenditure measurement limiting our ability to perform generalizations to large patient populations. 

Lastly, the strengths of this review are several. First, it adds to the clinical literature a summary of the different components of total energy expenditure except for resting energy expenditure, as this has been previously summarized and reported in the literature [13,14,24,25,26]. Second, it highlights the need for additional work that can determine the contribution of activity and thermic effect of feeding on total energy expenditure, especially during phases of recovery from acute and critical illness. Finally, this review reflects the wide variability and need for harmonization in how energy expenditure is evaluated and reported in the literature. 

## 5. Conclusions

This is one of the first studies that summarizes all components of total energy expenditure in different pediatric age groups in health and critical illness. This review emphasizes the scarcity of data on the contribution of growth and feeding to energy expenditure estimation in states of health, and the lack of data on total energy expenditure and its components in stages of acute recovery from critical illness. 

## Figures and Tables

**Table 1:** Characteristics of Included Studies (n = 134).

Characteristic	Summary
**Study Design**	
Cross-Sectional	127 (95%)
Longitudinal	7 (5%)
**Publication Years**	
1980–1990	5 (3%)
1991–2000	41 (31%)
2001–2010	45 (34%)
2011–2023	43 (32%)
**Median Age (months)**	114 (63.6, 156)
**Median Sample Size of Included Studies**	20 (12, 35)
**Disease States**	
Healthy Non-Obese	109 (81%)
Healthy Obese	20 (15%)
Healthy Recovering from Burns	1 (1%)
Critically Ill	4 (3%)
**Energy Expenditure Measurement Methods**	
Doubly Labelled Water	71 (53%)
Indirect Calorimetry	14 (10%)
Combined	48 (36%)

N (%), Median (Q1, Q3).

**Table 2:** Energy Expenditure Components Across Studies.

EE Component	Studies	Subjects	Measurements	Kcal/kg/day	Measurements	Kcal/day	Measurements
**AEE**	42	2706	4631				
**Age**							
Infants		78	78	15.0 (13.3, 17.7)	78		
Child		1053	1784	18.5 (17.3, 20.2)	73	1010 (403.5, 2880)	1711
Adolescent		890	1970	41.3 (18.3, 77.1)	90	2404 (761.3, 4140)	1880
Mixed		685	799	24.7 (20.6, 27.6)	79	498.5 (388, 2654)	720
**Condition**							
Healthy	42	2706	4631				
Critically Ill							
**TEF**	4	59	80				
**Age**							
Infants							
Child							
Adolescent		49	60			289.5 (222, 386)	60
Mixed		10	20			66.6 (63.4, 69.8)	20
**Condition**							
Healthy	4	59	80				
Critically Ill							
**GEE**	1	12	24				
**Age**							
Infants		12	24	26.3 (25.1, 27.5)	24		
Child							
Adolescent							
Mixed							
**Condition**							
Healthy	1	12	24				
Critically Ill							
**TEE**	124	7652	7750				
**Age**							
Infants		1036	1112	73.1 (67.0, 76.5)	891	583.2 (462.5, 1865.0)	221
Child		3424	3424	78.0 (66.0, 81.3)	589	1474 (1285.8, 1809.2)	2835
Adolescent		1843	1843	44.2 (41.8, 51.9)	177	2479 (2137.8, 2839.6)	1666
Mixed		1349	1371	69.3 (61.0, 72.9)	250	1932.5 (1713.8, 2313.5)	1121
**Condition**							
Healthy	119	7496	7594	76.2 (68.4, 79.1)	1805	1930.0 (1563.5, 2462.4)	5789
Critically Ill	5	156	156	52.1 (44.2, 57.7)	102	756.4 (484.3, 1160.5)	54

Infant (1 month–1 year), child (1 year–10 years), adolescent (>10 years). Studies included different age categories; therefore, the number of studies reporting on different age categories was not reported here. Critically ill patients had only total energy expenditure measured and in relatively small numbers. These results were not separated out. EE: Energy Expenditure, AEE: Activity-related energy expenditure, TEF: thermic effect of feeding, GEE: Growth energy expenditure, TEE: Total energy expenditure.

## Supplementary Materials

The following supporting information can be downloaded at: https://www.mdpi.com/article/10.3390/nu16162581/s1, Table S1: Study Details.

## Appendix A

**Table A1:** Search strategies. We used the following keywords and searched the following databases.

**EMBASE**		
	**Searches**	**Results**
1	energy expenditure.mp and exp energy expenditure	40,722
2	exp indirect calorimetry	3547
3	exp doubly labeled water technique/or double labelled water.mp	853
4	2 or 3	4341
5	1 and 4	2581
6	paediatric.mp or exp pediatrics	258,660
7	Limit 6 to (human and english language)	203,546
8	5 and 7	24
9	exp adolescent/or adolescent.mp	1,968,375
10	neonatal.mp	354,562
11	infant.mp or exp infant	1,419,064
12	9 or 10 or 11	3,262,912
13	5 and 12	244
**OVID MEDLINE**		
	**Searches**	**Results**
1	energy expenditure.mp	26,599
2	indirect calorimetry.mp or exp Calorimetry, Indirect/	8044
3	doubly labelled water.mp	457
4	doubly labeled water.mp	1059
5	3 or 4	1504
6	2 or 5	9203
7	1 and 6	5313
8	Limit 7 to (English language and humans and (* “all infant (birth to 23 months)” or “all child (0 to 18 years)”))	1411
**CINAHL**		
	**Searches**	**Results**
S1	(MH “Energy Metabolism+”) OR “Energy Expenditure”	29,736
S2	“indirect calorimetry”	2133
S3	(MH “Doubly Labeled Water Technique”) OR “doubly labelled water”	533
S4	S2 OR S3	2614
S5	S1 AND S4	2018
S6	“paediatric”	28,112
S7	“pediatric”	180,805
S8	“neonatal”	81,173
S9	(MH “Infant”) OR “infant”	315,650
S10	“adolescent”	100,222
S11	S6 OR S7 OR S8 OR S9 OR S10	585,841
S12	S5 AND S11	234

## References

[1] Pinheiro Volp A.C., Esteves de Oliveira F.C., Duarte Moreira Alves R., Esteves E.A., Bressan J. (2011). Energy expenditure: Components and evaluation methods. Nutr. Hosp..

[2] Butte N.F., Wong W.W., Ferlic L., Smith E.O., Klein P.D., Garza C. (1990). Energy expenditure and deposition of breast-fed and formula-fed infants during early infancy. Pediatr. Res..

[3] Luo Z.C., Cheung Y.B., He Q., Albertsson-Wikland K., Karlberg J. (2003). Growth in early life and its relation to pubertal growth. Epidemiology.

[4] Wiskin A.E., Davies J.H., Wootton S.A., Beattie R.M. (2011). Energy expenditure, nutrition and growth. Arch. Dis. Child..

[5] Donahoo W.T., Levine J.A., Melanson E.L. (2004). Variability in energy expenditure and its components. Curr. Opin. Clin. Nutr. Metab. Care.

[6] Hills A.P., Mokhtar N., Byrne N.M. (2014). Assessment of physical activity and energy expenditure: An overview of objective measures. Front. Nutr..

[7] Pontzer H., Yamada Y., Sagayama H., Ainslie P.N., Andersen L.F., Anderson L.J., Arab L., Baddou I., Bedu-Addo K., Blaak E.E. (2021). Daily energy expenditure through the human life course. Science.

[8] Westerterp K.R. (2013). Physical activity and physical activity induced energy expenditure in humans: Measurement, determinants, and effects. Front. Physiol..

[9] Davies P.S., Wells J.C., Hinds A., Day J.M., Laidlaw A. (1997). Total energy expenditure in 9 month and 12 month infants. Eur. J. Clin. Nutr..

[10] Brochu P., Ducre-Robitaille J.F., Brodeur J. (2006). Physiological daily inhalation rates for free-living individuals aged 1 month to 96 years, using data from doubly labeled water measurements: A proposal for air quality criteria, standard calculations and health risk assessment. Hum. Ecol. Risk Assess..

[11] Mtaweh H., Tuira L., Floh A.A., Parshuram C.S. (2018). Indirect Calorimetry: History, Technology, and Application. Front. Pediatr..

[12] Tatucu-Babet O.A., Nguo K., Lambell K.J., Romero L., Earthman C.P., Ridley E.J. (2022). Doubly labelled water for determining total energy expenditure in adult critically ill and acute care hospitalized inpatients: A scoping review. Clin. Nutr..

[13] Jotterand Chaparro C., Moullet C., Taffe P., Depeyre J.L., Perez M.H., Longchamp D., Cotting J. (2018). Estimation of Resting Energy Expenditure Using Predictive Equations in Critically Ill Children: Results of a Systematic Review. J. Parenter. Enter. Nutr..

[14] Jotterand Chaparro C., Taffe P., Moullet C., Laure Depeyre J., Longchamp D., Perez M.H., Cotting J. (2017). Performance of Predictive Equations Specifically Developed to Estimate Resting Energy Expenditure in Ventilated Critically Ill Children. J. Pediatr..

[15] Cuthbertson D. (1970). Intensive-care-metabolic response to injury. Br. J. Surg..

[16] Joffe A., Anton N., Lequier L., Vandermeer B., Tjosvold L., Larsen B., Hartling L. (2009). Nutritional support for critically ill children. Cochrane Database Syst. Rev..

[17] Joffe A., Anton N., Lequier L., Vandermeer B., Tjosvold L., Larsen B., Hartling L. (2016). Nutritional support for critically ill children. Cochrane Database Syst. Rev..

[18] Mtaweh H., Garros C., Ashkin A., Tuira L., Allard J.P., Pencharz P., Pullenayegum E., Joffe A., Parshuram C.S. (2020). An Exploratory Retrospective Study of Factors Affecting Energy Expenditure in Critically Ill Children. JPEN J. Parenter. Enter. Nutr..

[19] Haas V.K., Gaskin K.J., Kohn M.R., Clarke S.D., Muller M.J. (2010). Different thermic effects of leptin in adolescent females with varying body fat content. Clin. Nutr..

[20] Maffeis C., Schutz Y., Micciolo R., Zoccante L., Pinelli L. (1993). Resting metabolic rate in six- to ten-year-old obese and nonobese children. J. Pediatr..

[21] Framson C.M., LeLeiko N.S., Dallal G.E., Roubenoff R., Snelling L.K., Dwyer J.T. (2007). Energy expenditure in critically ill children. Pediatr. Crit. Care Med..

[22] Torun B. (2005). Energy requirements of children and adolescents. Public Health Nutr..

[23] Kim N., Park J. (2023). Total energy expenditure measured by doubly labeled water method in children and adolescents: A systematic review. Clin. Exp. Pediatr..

[24] Mtaweh H., Soto Aguero M.J., Campbell M., Allard J.P., Pencharz P., Pullenayegum E., Parshuram C.S. (2019). Systematic review of factors associated with energy expenditure in the critically ill. Clin. Nutr. ESPEN.

[25] Macena M.L., Paula D., da Silva Junior A.E., Praxedes D.R.S., Pureza I., de Melo I.S.V., Bueno N.B. (2022). Estimates of resting energy expenditure and total energy expenditure using predictive equations in adults with overweight and obesity: A systematic review with meta-analysis. Nutr. Rev..

[26] Chima L., Mulrooney H.M., Warren J., Madden A.M. (2020). A systematic review and quantitative analysis of resting energy expenditure prediction equations in healthy overweight and obese children and adolescents. J. Hum. Nutr. Diet..

