# Supplementary material for: Components of Total Energy Expenditure in Healthy and Critically Ill Children: A Comprehensive Review
Source: Nutrients. 2024 Aug 6;16(16):2581. doi: 10.3390/nu16162581 (PMC11357425; doi:10.3390/nu16162581)
Supplement: Supplementary file 1 [file nutrients-16-02581-s001.zip › nutrients-3084691-supplementary.pdf]

Table S1. Study Details.

Author	Year	Title	Objective	Study Design	Population and Measurements	EE Method	EE Components Studied
Abbott[1]	2004	Habitual physical activity and physical activity intensity: their relation to body composition in 5.0–10.5-y-old children	To examine the relationship between physical activity, minutes spent in various intensities of physical activity, EE, and body composition.	Prospective, cross-sectional	Healthy children aged 5–10.5 years measured over 10 days.	DLW	TEE
Arvidsson[2]	2005	Physical activity questionnaire for adolescents validated against doubly labelled water	To validate a physical activity questionnaire for adolescents (PAQA) adapted from the International Physical Activity Questionnaire (IPAQ).	Prospective, cross-sectional	Healthy Swedish adolescents aged 15.7 ± 0.4 years (mean ± SD).<br>TEE was measured by DLW over 14 days.	DLW	TEE
Arvidsson[3]	2009	Free-living energy expenditure in children using multi-sensor activity monitors	To improve the EE algorithm of the activity monitor ActiReg, and to validate ActiReg and the activity monitor SenseWear. IC was used as reference.	Prospective, cross-sectional	Healthy adolescents aged 11–13 years, measured on a treadmill walking and running.<br>TEE was measured by DLW under free-living conditions for 14 days. REE was measured by an Oxycon Mobile IC.	DLW<br>IC (Oxycon Mobile)	TEE<br>REE
Backlund[4]	2010	Validity of Armband Measuring Energy Expenditure in Overweight and Obese Children	To examine the ability of the SenseWear Pro2 Armband (SWA) to assess EE in by comparison with TEE measured using DLW, and to examine which software version, Interview Professional 5.1 or Sensewear Professional 6.0, is the most appropriate for use together with the SWA.	Interventional	Healthy, free-living overweight or obese children aged 8–11 years measured during 2-week period.	DLW	TEE
Ball[5]	2001	Total energy expenditure, body fatness, and physical activity in children aged 6–9 y	To measure TEE, determine the proportion of TEE related to physical activity, and investigate relations between measures of physical activity and body fatness (including sex differences).	Prospective, cross-sectional	Healthy children aged 7.8 ± 0.9 years (mean ± SD) over 10 days.	DLW	TEE
Bandini [6]	1990	Energy expenditure in obese and nonobese adolescents	To determine whether EE differences exist after obesity develops.	Prospective, cross-sectional	Healthy obese and non-obese adolescents aged 12 to 18 years, with TEE measured over 2 weeks, and BMR measured for 30 min the morning after admission. During admission, subjects were instructed on food portion sizes.	DLW<br>IC	TEE<br>BMR
Bandini [7]	2002	Relation of body composition, parental overweight, pubertal stage, and race-ethnicity to energy expenditure among premenarcheal girls	To determine whether differences in EE in premenarcheal girls are related to pubertal stage, race-ethnicity, or parental weight status.	Prospective, cross-sectional	Healthy non-obese, premenarcheal girls aged 8–12 years. TEE was measured by DLW during a 2-week period. REE was measured by IC for 30 min after an overnight fast, proceeded by a 30 min rest period.	DLW<br>IC	TEE<br>REE
Bandini [8]	1989	Energy expenditure during carbohydrate	To measure EE.	Interventional	Healthy non-obese and obese adolescents aged 12–19 years, during periods of weight	DLW<br>IC	TEE<br>BMR

		overfeeding in obese and nonobese adolescents			maintenance and 2 weeks of carbohydrate overfeeding. Energy intake was 1.61xBMR during maintenance and 2.45xBMR during overfeeding. The maintenance period was 3–4 days in four subjects, and 8–9 days in the remaining nine subjects. A 13–15-day overfeeding period followed the maintenance period.<br><br>TEE was measured by DLW over 14 days. BMR was measured by IC after one or two overnight visits before admission. TEF was calculated by dividing the increase in EE by the calories in the test meal.	C (TEF = increase in EE/calories in test meal)	TEF
Bell[9]	2010	Energy expenditure and physical activity of ambulatory children with cerebral palsy and of typically developing children	To investigate the components of TEE in children with cerebral palsy versus typically developing children and to determine what effect the higher EE during walking has in ambulatory children with CP on PAL and TEE.	Prospective, cross-sectional	Healthy, typically developing children aged 5–12 years. Children with Cerebral Palsy were excluded. The activity used for AEE was walking at 72 ± 0.8 m/min (mean ± SD).<br><br>TEE was measured by DLW. REE was measured by IC. AEE was calculated as 0.09TEE − REE.	DLW<br>IC<br>C (AEE = 0.09TEE − REE)	TEE<br>REE<br>AEE
Bitar[10]	1999	Variations and determinants of energy expenditure as measured by whole-body indirect calorimetry during puberty and adolescence	To measure EE.	Prospective, cross-sectional	Healthy children and adolescents aged 10–16 years, measured continuously over 24 h using whole-body indirect calorimeters during four 15 min periods of exercise (0930, 1100, 1500, 1730), at four intensities on a cycle ergometer (40% peak VO_2, 50% peak VO_2, 30% peak VO_2, 60% peak VO_2)	IC	TEE<br>REE<br>AEE
Bornhorst[11]	2014	Validity of 24-h recalls in (pre-)school aged children: comparison of proxy-reported energy intakes with measured energy expenditure	Evaluated the validity of 24 h recalls of energy intake compared to measured EE.	Prospective, cross-sectional	Healthy 4–10-year-olds from Belgium, Sweden, and Spain.<br><br>TEE was measured by DLW over 9 days.	DLW	TEE
Brage[12]	2021	Descriptive epidemiology of energy expenditure in the UK: Findings from the National Diet and Nutrition Survey 2008-15	To describe TEE of DLW-subsample participants from the National Diet and Nutrition Survey (NDNS) in the UK aged 4–91 years, recruited between 2008 and 2015.	Prospective, cross-sectional	Healthy individuals aged 4–91 years (we excluded individuals older than 18 years old). TEE was measured by DLW over 10 days.	DLW	TEE
Brandes [13]	2018	Energy Cost of Common Physical Activities in Preschoolers	To determine the PAEE and to compare it with the Compendium of Energy Expenditure for Youth (CEEY).	Prospective, cross-sectional	Healthy children aged 4.8 ± 0.8 years (mean ±SD), measured during completion of 13 common physical activities including **Indoor Activities** (drawing, building, hide-and-seek, cars, dolls) and **Outdoor Activities** (tag, tricycle, climbing, swinging, walking, walking fast, jogging). Nine indoor and outdoor activities were assigned, of which five were light to vigorous intensity. Children then picked four more activities for themselves, for a total of 75 min for all activities and preparation including getting dressed.<br><br>RMR was measured as the minimum of a rolling 1 min mean during supine rest. EE during activity was measured by a portable,	IC<br>C (AEE = $EE_{activity}$ − ($EE_{eactivity}$/AME)	RMR<br>AEE

					open-circuit IC (MetaMax 3B). We calculated AEE from AME as $E_{activity} - (EE_{activity}/AME)$.		
Bratteby [14]	1997	A 7-day activity diary for assessment of daily energy expenditure validated by the doubly labelled water method in adolescents	To validate the use of an activity diary and predict BMR for the assessment of daily TEE and PAL.	Prospective, cross-sectional	Healthy 15-year-old adolescents living in Sweden.<br><br>TEE was measured by DLW over 14 days. REE was measured by IC while lying supine for 15–30 min.	DLW<br>IC (Oxycon Sigma)	TEE<br>REE
Bratteby [15]	1998	Total energy expenditure and physical activity as assessed by the doubly labeled water method in Swedish adolescents in whom energy intake was underestimated by 7-d diet records	To investigate whether assessment of TEE and PAL by DLW and IC and estimation of energy intake by a 7-day diet record would indicate physical inactivity. Equation.	Prospective, cross-sectional	Healthy adolescents aged 15 years in Uppsala, Sweden.<br><br>TEE was measured by DLW over 14 days. BMR was measured by open-circuit Oxycon Sigma IC, fasted, after which a light breakfast was served.<br>AEE was calculated as TEE − BMR during free-living activities.	DLW<br>IC<br>C (AEE = TEE − BMR)	TEE<br>BMR<br>AEE
Burrows[16]	2013	Comparison and validation of child versus parent reporting of children's energy intake using food frequency questionnaires versus food records: Who's an accurate reporter?	To compare the accuracy of reporting for a child's energy intake from an FFQ completed independently by the mother, father, and child in comparison to a weighed food record (WFR) and measure by DLW.	Interventional	Healthy-weight children aged 8–11 years with two eligible caregivers (residing with the child) from the Hunter region, in NSW Australia. Usual dietary patterns and physical activity habits were maintained.<br>TEE was measured by DLW over 10 days.	DLW	TEE
Butte[17]	1990	Energy expenditure and deposition of breast-fed and formula-fed infants during early infancy	To measure and calculate EE.	Prospective, cross-sectional	Healthy breast-fed and formula-fed infants at 1 and 4 months of age measured at post-prandial intervals 0 to 2 h, 2 to 3 h, and 3 to 4 h<br><br>TEE was measured by DLW over 14 days. SMR was measured by IC. AEE was calculated as TDEE − SMR.	DLW<br>IC<br>C (AEE = TDEE − SMR)	TEE<br>AEE
Butte[18]	2001	Energy requirements derived from total energy expenditure and energy deposition during the first 2 y of life	To measure EE.	Prospective, cross-sectional	Healthy, full-term, infants at ages 3, 6, 9, 12, 18, and 24 months, measured over 10 days.	DLW	TEE
Butte[19]	2014	Prediction of energy expenditure and physical activity in preschoolers	To re-examine dietary reference intakes for preschool-aged children and compare to measured TEE.	Prospective, cross-sectional	Healthy, normal weight preschool-age children (3–5 years) measured over 7 days.	DLW	TEE
Butte[20]	2016	Role of physical activity and sleep duration in growth and body composition of preschool-aged children	To measure EE, body size, and composition and determine whether physical activity components, sleep, and TEE predict 1-year changes in body size and composition.	Prospective, longitudinal	Healthy children aged 3–5 years living in the greater Houston area. TEE was measured under free-living conditions by DLW over 7 days, and repeated 1 year later.	DLW	TEE
Butte[21]	2010	Validation of cross-sectional time series and multivariate adaptive regression splines models for	To validate cross-sectional time series (CSTS) and multivariate adaptive regression splines (MARS) models based on observable	Prospective, cross-sectional	Healthy children aged 5–18 years. TEE was measured by DLW under free-living conditions over 7 days	DLW	TEE

		the prediction of energy expenditure in children and adolescents using doubly labeled water	participants characteristics, heart rate, and accelerometer counts for prediction of minute-by-minute EE, and hence 24 h TEE against a 7-day DLW method.				
Byun[22]	2016	Energy Expenditure of Daily Living Activities in 3- to 6-Year-Old Children	To examine activity EE.	Interventional	Healthy children aged 3–6 years, measured during **Sedentary activities** (TV watching lying down, TV watching sitting), and **Light to Moderate Activities** (playing with toys including blocks/cars/puzzles, walking/exploring and scavenger hunt) or **Moderate to Hard Activities** (soccer/running, basketball/throwing).<br><br>We used the sedentary activities as "REE", and used the activity with the lowest measured EE (TV watching lying down) as our "REE" during AEE calculations.<br><br>EE was measured by IC (The Oxycon Mobile).<br>We calculated AEE as $EE_{activity}$ − ($EE_{TV\ Watching\ Lying\ Down}$).	IC<br>C (AEE = $EE_{activity}$ − ($EE_{TV\ Watching\ Lying\ Down}$))	REE<br>AEE
Calabro [23]	2013	Validation of pattern-recognition monitors in children using doubly labeled water	To validate SenseWear Armband monitors under free-living conditions in youth populations.	Prospective, cross-sectional	Healthy 10–16-year-old adolescents with various ethnic backgrounds (76% Caucasian, 14% Hispanic, 10% Asian), and body types (16.7% at risk for overweight, 3.3% overweight, 6.7% underweight). TEE was measured by DLW over 14 days. REE was measured by IC on day 7 after an overnight fast. AEE was calculated as 0.9TEE − RMR.	DLW<br>IC (True One 2400 Metabolic Measurement System)<br>C (AEE = 0.9TEE − RMR)	TEE<br>RMR<br>AEE
Campbell[24]	2012	The Actiheart in adolescents: a doubly labelled water validation	To investigate the validity of the Actiheart device for estimating PAEE.	Prospective, cross-sectional	Healthy adolescents aged 17.50 ± 0.62 years (mean ± SD), from two secondary and one postsecondary institutions in Ontario.<br>TEE was measured by DLW over 9 days.	DLW	TEE
Carter[25]	2008	An investigation of a novel three-dimensional activity monitor to predict free-living energy expenditure	To assess the capability of the 3dNX accelerometer to predict EE in two separate, free-living cohorts.	Prospective, cross-sectional	Healthy adolescents and young adults aged 16 ± 3 years (mean ± SD). TEE was measured by DLW over 10 days.	DLW	TEE
Collins[26]	2013	Comparison of energy intake in toddlers assessed by food frequency questionnaire and total energy expenditure measured by the doubly labeled water method	To evaluate the accuracy of toddler energy intake (EI), estimated using the Australian Child and Adolescent Eating Survey (ACAES) food frequency questionnaire (FFQ) by parent report compared with a weighed food record (WFR) and TEE measured by DLW.	Interventional	Healthy children aged approximately 3 years, from the Hunter Region of New South Wales, Australia. The children fasted overnight before the first visit, but then maintained usual eating habits thereafter.<br><br>TEE was measured by DLW over 10 days.	DLW	TEE
Corder[27]	2009	Is it possible to assess free-living physical activity and energy expenditure in young people by self-report?	To assess the validity and reliability of four self-reports to assess PAEE and time spent at moderate and vigorous intensity physical activity (MVPA).	Prospective, cross-sectional	Healthy, young British people 4–17 years of age. TEE was measured by DLW over 11 days.	DLW	TEE
Corder[28]	2010	Physical Activity Energy Expenditure of Adolescents in India	To assess whether PAEE could be accurately estimated using an	Prospective, cross-sectional	Healthy Indian adolescents aged 15.8 ± 0.59 years old (mean ±SD).	DLW	TEE

			accelerometer and questionnaire.		TEE was measured under free-living conditions over 7 days.		
Dabare[29]	2023	Prediction Equation for Physical Activity Energy Expenditure in 11–13-Year-Old Sri Lankan Children	To develop a regression equation to predict PAEE using accelerometry.	Prospective, cross-sectional	Healthy adolescents aged 11–13 years. TEE was measured by DLW over 10 days.	DLW	TEE
Davidsson[30]	2016	Total Energy Expenditure in Obese Kuwaiti Primary School Children Assessed by the Doubly-Labeled Water Technique	To assess body composition and TEE of obese Kuwaiti children.	Prospective, cross-sectional	Healthy, obese, 7–9-year-old Kuwaiti children, with TEE measured by DLW over 14 days.	DLW	TEE
Davies[31]	1988	Energy expenditure in early infancy	To measure EE.	Prospective, longitudinal	Healthy, normal, full-term infants at or close to 6 weeks, 2 months, and 6 months of age measured over 7 days.	DLW	TEE
Davies[32]	1997	Total energy expenditure in 9 month and 12 month infants	To measure EE.	Prospective, cross-sectional	Healthy 9- and 12-month-old infants measured over 10 days.	DLW	TEE
Davies[33]	1991	Energy expenditure in early infancy and later body fatness	To measure EE at 12 weeks. These data were related to Quetelet's index and the sum of the triceps and sub-scapular skinfold measurements at 9 months and 2 years of age.	Prospective, cross-sectional	Healthy, full-term infants at 12 weeks of age. TEE was measured by DLW over 7 days.	DLW	TEE
Davies[34]	1994	Total energy expenditure and energy intake in the pre-school child: a comparison	To measure energy intake and EE and compare them.	Prospective, cross-sectional	Healthy children aged 1.5–4.5 years in Cambridgeshire, England. TEE was measured by DLW over 10 days.	DLW	TEE
Davies[35]	1995	Physical activity and body fatness in pre-school children	To investigate the relationship between levels of physical activity and body fatness.	Prospective, cross-sectional	Healthy preschool children aged 1.5–4.5 years, recruited from towns in southeast England. TEE was measured by DLW over 10 days.	DLW	TEE
Davies[36]	1995	Energy expenditure in children aged 1.5 to 4.5 years: a comparison with current recommendations for energy intake	To compare measurements EE with current recommendations for energy intake.	Prospective, cross-sectional	Healthy preschool children aged 1.5–4.5 years, recruited from towns in southeast England. TEE was measured by DLW over 10 days.	DLW	TEE
de Bruin[37]	1997	Energy utilization and growth in breast-fed and formula-fed infants measured during the first year of life	To determine energy requirements for male and female breast-fed and formula-fed infants using TEE and energy deposition derived from total body fat (TBF) and fat-free mass (FFM) gain.	Prospective, cross-sectional	Healthy full-term, breast-fed, and formula-fed infants 4 months or older, measured over 8 days.	DLW	TEE
DeLany [38]	2002	Energy expenditure in preadolescent African American and white boys and girls: the Baton Rouge Children's Study	To determine the relations between race, sex, body fat, and EE. AEE = TDEE − RMR − TEF.	Prospective, cross-sectional	Healthy, pre-adolescent children at Tanner stages 1 and 2 only, with a similar number of African American and white children. TEE was measured by DLW over 7 days. RMR was measured by IC for 30 min, followed by a test meal, when metabolic rate was measured for 3 h to estimate TEF (%meal). We did not use this TEF measurement.	DLW<br>IC<br>C (AEE = TDEE − RMR − TEF)	TEE<br>RMR<br>AEE

DeLany [39]	2004	Energy expenditure in African American and white boys and girls in a 2-y follow-up of the Baton Rouge Children's Study	To determine the relations between race, sex, Tanner stage, and EE. Follow up study from 2002.	Prospective, cross-sectional	Healthy obese and lean African American and white adolescents aged 12.7 ± 0.1 years (mean ± SD).<br>TEE was measured by DLW over 7 days. RMR was measured by IC. AEE was calculated as TDEE − RMR − TEF.<br>TEF was excluded as it was measured as % meal.	DLW<br>IC<br>C (AEE = TDEE − RMR − TEF)	TEE<br>REE<br>AEE
Delisle Nystrom[40]	2017	Validation of an Online Food Frequency Questionnaire against Doubly Labelled Water and 24 h Dietary Recalls in Pre-School Children	To determine the validity of wrist-worn ActiGraph wGT3x-BT for estimation of AEE in preschool children, to measure TEE, and to assess wear compliance.	Prospective, cross-sectional	Healthy Swedish preschool children 5.5 ± 0.2 years (mean ± SD) measured over 7 days.	DLW	TEE
Dugas[41]	2008	Very low levels of energy expenditure among pre-adolescent Mexican-American girls	To measure EE.	Prospective, cross-sectional	Healthy Mexican American and European American children aged 6–10 years. TEE was measured by DLW over either 7 or 14 days. REE was measured by IC after an overnight fast.	DLW<br>IC<br>C (AEE = 0.94TEE − REE)	TEE<br>REE<br>AEE
Dutman [42]	2011	Validation of an FFQ and options for data processing using the doubly labelled water method in children	To determine the validity of a food frequency questionnaire (FFQ) by comparing with TEE measured by DLW.	Prospective, cross-sectional	Healthy children aged 4–6 years measured over 15 days.	DLW	TEE
Ekelund [43]	2001	Physical activity assessed by activity monitor and doubly labeled water in children	To validate the computer science and applications (CSAs) activity monitor for assessment of total physical activity during, to develop predictive equations for TEE and AEE from activity counts and anthropometric variables.	Prospective, cross-sectional	Healthy children aged 9 years, measured for 14 days.	DLW	TEE
Ekelund [44]	2002	Physical activity but not energy expenditure is reduced in obese adolescents: a case-control study	To test the hypothesis that the intensity and duration of physical activity differ between obese and non-obese adolescents.	Prospective, cross-sectional	Healthy obese adolescents aged 14–19 years with a body mass index > 30, matched to controls (BMI < 27) from the same school in Sweden. Subjects were served a light breakfast, and then performed an exercise test (walking on a treadmill at 4 km/h and 6 km/h for 5 min each). They were measured for 15 days.<br><br>TEE was measured by DLW. REE was measured by Douglas Bags IC. AEE was calculated (TEE − (REE + 0.1TEE)) during activities of free-living.	DLW<br>Douglas Bags IC<br>C (AEE = TEE − (REE +0.1TEE))	TEE<br>REE<br>AEE
Ekelund [45]	2004	Body movement and physical activity energy expenditure in children and adolescents: how to adjust for differences in body size and age	To examine whether physical activity expressed as body movement (accelerometer counts) differ from PAEE as a function of body size and age.	Prospective, cross-sectional	Healthy children aged 9.6 ± 0.3 years (mean ± SD) and adolescents 17.6 ± 1.5 years (mean ± SD).<br><br>TEE was measured by DLW over 10–14 days. REE was measured by IC after an overnight fast.<br>AEE was calculated as 0.9TEE − REE during free-living activities.	DLW<br>IC<br>C (AEE = 0.9TEE − REE)	TEE<br>REE<br>AEE
Eliakim[46]	2000	Training, muscle volume, and energy expenditure in nonobese American girls	To analyze the effect of a 5-week school-based program of endurance exercise in healthy, non-obese, prepubertal girls.	Interventional	Healthy children aged 9.17 ± 0.10 years (mean ± SD) randomized to a training group (2 × 45 min endurance training = running, jumping, aerobic dance, age-appropriate competitive sport including basketball and soccer) and	DLW	TEE

				control group (45 min science programming in physiology). TEE was measured by DLW for a 10-day period beginning of week 3 of the protocol.		
Elliot[47]	2014	Accuracy of Self-Reported Physical Activity Levels in Obese Adolescents	To investigate if obese adolescents could accurately report their physical activity levels using self-reported diaries.	Prospective, cross-sectional	Healthy obese adolescents 10–17.99 years of age.<br>TEE was measured by DLW over 10 days. REE was measured by IC with the subject resting supine after an overnight fast. AEE was calculated as TEE − REE, with active behaviour defined as any activity in which the MET value ranged from 3.5 to 5.0.	DLW<br>IC<br>C (AEE = TEE − REE)	TEE<br>REE<br>AEE
Fisher[48]	2000	Influence of body composition on the accuracy of reported energy intake in children	To evaluate children's dietary reporting accuracy as a function of their relative weight, body composition, and macronutrient intake.	Prospective, cross-sectional	Healthy 4–11-year-old children and adolescents.<br>TEE was measured by DLW under free-living conditions over 14 days.	DLW	TEE
Fontvieille[49]	1993	Daily Energy Expenditure Measured by Doubly Labelled Water		Prospective, cross-sectional	Healthy white children aged 5 years.<br>TEE was measured by DLW over 7 days. RMR was measured with the Deltatrac metabolic cart IC for 20 min, after the child had been lying in bed for 10 min, on the first and second hospital admissions. AEE was calculated as TEE − RMR − 0.1TEE during activities of free-living.	DLW<br>IC<br>C (AEE = TEE − RMR − 0.1TEE)	TEE<br>RMR<br>AEE
Franks[50]	2005	Habitual physical activity in children: the role of genes and the environment	To quantify the genetic and environmental influences on PAEE.	Prospective, cross-sectional	Healthy, sex-concordant dizygotic and monozygotic twin pairs aged 4–10 years.<br>TEE was measured by DLW over 7 days. RMR was measured by IC for 20 min after resting in bed for 10 min. AEE was calculated as TEE − (RMR + 0.1TEE) during activities of free-living.	DLW<br>IC (DeltaTrac Metabolic Monitor)<br>C (AEE = TEE − (RMR + 0.1TEE))	TEE<br>RMR<br>AEE
Gibson[51]	2013	Aerobic Capacity, Activity Levels and Daily Energy Expenditure in Male and Female Adolescents of the Kenyan Nandi Sub-Group	To measure aerobic capacity and EE.	Prospective, cross-sectional	Healthy, habitually active adolescents of the Kenyan Nandi Sub-Group with males aged 13.9 ± 1.6 years and females aged 13.9 ± 1.2 years (mean ± SD). TEE was measured by DLW over 7 days.	DLW	TEE
Gondolf[52]	2012	Validation of a pre-coded food record for infants and young children	To assess the validity of a 7-day pre-coded food record (PFR) and weighed-food record (WFR) methods against metabolizable energy intake measured by DLW.	Interventional	Healthy 9- and 36-month-old infants and children randomized to either 7-days PFR or WFR methods.<br>TEE was measured by DLW over 7 days.	DLW	TEE
Goran[53]	1997	Physical activity related energy expenditure and fat mass in young children	To examine whether body fat content in prepubertal children is influenced by PAEE and/or more qualitative aspects of physical activity.	Prospective, cross-sectional	Prepubertal children aged 6.3 ± 0.9 years (mean ±SD), as a follow-up study. TEE was measured by DLW under free-living conditions over 14 days.<br>Postprandial RMR was measured by a portable Deltatrac metabolic monitor (IC), 2–3 h after the subjects consumed a normal breakfast at home. AEE was calculated as TEE − postprandial RMR.	DLW<br>IC (Deltatrac metabolic monitor)<br>C(AEE = TEE − postprandial RMR)	TEE<br>RMR<br>AEE
Goran[54]	1993	Total energy expenditure in 4- to 6-yr-old children	Examined the components of daily energy expenditure in a group of 30 children, characterized for body	Prospective, cross-sectional	Healthy Caucasian 4–6-year-olds in Burlington, Vermont.	DLW<br>IC<br>C (AEE = TEE − REE)	TEE<br>REE<br>AEE

			weight, height, heart rate, and body composition from bioelectrical resistance		TEE was measured under free-living conditions by DLW over 14 days. REE was measured by IC, after subjects had eaten their usual breakfast, as subjects lay still and watched video cartoons (TEF is accounted for in this measurement). AEE was calculated as TEE − REE during activities of free-living.		
Goran[55]	1998	Developmental Changes in Energy Expenditure and Physical Activity in Children: Evidence for a Decline in Physical Activity in Girls Before Puberty	To examine individual changes in EE and physical activity during pubertal growth.	Prospective, longitudinal	Healthy girls aged 5.5 ± 0.9 years at baseline (mean ± SD), and boys aged 5.3 ± 0.9 years at baseline, measured over 5 years at year 1, 2, and 5.<br><br>TEE was measured by DLW over 14 days. REE was measured under postprandial conditions (2–3 h after children consumed their usual breakfast at home) 14 days apart by IC (Deltatrac metabolic monitor). AEE was calculated as TEE − postprandial REE.	DLW<br>IC (Deltatrac metabolic monitor)<br>C (AEE = TEE − postprandial REE)	TEE<br>REE<br>AEE
Goran[56]	1995	Energy expenditure and body fat distribution in Mohawk children	Measure EE and body fat distribution.	Prospective, cross-sectional	Healthy Mohawk and Caucasian children aged 4–7 years from upstate New York and Burlington, VT, respectively.<br>TEE was measured by DLW over 14 days. REE was measured under postprandial conditions, 14 days apart by IC (Deltatrac metabolic monitor). AEE was calculated as TEE − postprandial REE.	DLW<br>IC<br>C (AEE = TEE − REE)	TEE<br>REE<br>AEE
Gregory [57]	1992	Effects of oral testosterone undecanoate on growth, body composition, strength and energy expenditure of adolescent boys	To determine the effect of 3 months of daily 40 mg testosterone undecanoate on growth, body composition, hand grip, and quadricep muscle strength and total free-living daily EE in boys with constitutionally delayed puberty.	Interventional	Healthy boys with constitutionally delayed puberty aged 13.2 ± 1.6 years (mean ± SD), grouped into a placebo group and a treatment group, treated with testosterone undecanoate. TEE was measured by DLW over 14 days.	DLW	TEE
Hallal[58]	2013	Energy expenditure compared to physical activity measured by accelerometry and self-report in adolescents: a validation study	To compare physical activity measured by accelerometer and questionnaire against TEE and physical activity energy expenditure (PAEE) estimated by DLW.	Prospective, cross-sectional	Healthy 13-year-old adolescents living in Brazil, measured over a 10-day period.	DLW	TEE
Henriksson[59]	2014	Evaluation of Actiheart and a 7-d activity diary for estimating free-living total and activity energy expenditure using criterion methods in 1.5- and 3-year-old children	To evaluate the capacity of Actiheart and mean activity accounts (mAc) and activity diary measurements to measure TEE, to compare with gold standard DLW and IC.	Prospective, longitudinal	Healthy children at 1.5 year and 3 years of age, measured over 14 days.<br><br>TEE measured by DLW. SMR was measured by IC (Deltatrac metabolic monitor) during a midmorning nap. AEE was calculated as TEE − SMR.	DLW<br>IC (Deltatrac Metabolic Monitor)<br>C (AEE = TEE − SMR)	TEE<br>REE<br>AEE
Henriksson[60]	2015	A New Mobile Phone-Based Tool for Assessing Energy and Certain Food Intakes in Young Children: A Validation Study	To compare energy intake by means of the Tool for Energy Balance in Children (TECH) with TEE measured using the DLW technique, and to compare intakes of fruits and berries, vegetables, juice, and sweetened beverages assessed by means of TECH with intakes obtained using	Interventional	Healthy, Swedish 3-year-olds, whose meals were assessed and monitored using TECH. TEE was measured by DLW over 14 days.	DLW	TEE

		a web-based frequency questionnaire.				
Hoffman[61]	2000	Energy expenditure of stunted and nonstunted boys and girls living in the shantytowns of Sao Paulo, Brazil	To test the hypothesis that stunted children have lower EE and therefore a higher risk of obesity versus non-stunted children.	Prospective, cross-sectional	Healthy stunted and non-stunted children from Brazil, aged 8–11 years with similar weight-for-height.<br>TEE was measured by DLW over a 7-day period. REE was measured three times (15 min each for 2, 30 min for 1), using IC after a ~12 h overnight fast with subjects rested supine in bed.	DLW<br>DeltaTrac IC	TEE<br>REE
Hoos[62]	2003	Physical activity level measured by doubly labeled water and accelerometry in children	To investigate the use of a triaxial accelerometer (Tracmor2) to measure physical activity in children, and to measure TEE.	Prospective, cross-sectional	Healthy children aged 6.9 ± 2.2 years (mean ± SD) measured over 2 weeks.	DLW	TEE
Horswill[63]	1995	Energy expenditure in adolescents during low intensity, leisure activities	To determine the impacts of low versus high levels of activity on EE.	Prospective, cross-sectional	Healthy adolescents aged during free-living activities (TV viewing, playing a stringed instrument, and walking at 40% of peak VO_2). Subjects were measured 2–3 h after their last meal and were requested to maintain a similar diet on each day prior to test session. EE during activities was measured by IC, every 15–20 min (three times per exercise session). We used EE during TV Viewing as our "REE" for AEE calculations.<br><br>AEE was calculated as $EE_{activity}$ − $EE_{TV\ Watching}$.	C (AEE = $EE_{activity}$ − $EE_{TV\ Watching}$)	AEE
Hwang[64]	2019	Exploring Energy Expenditure and Body Movement of Exergaming in Children of Different Weight Status	To examine the differences in EE and bodily movement among children of different weight status during exergames that varied in mode and intensity.	Prospective, cross-sectional	Healthy overweight, obese, and normal weight 8–12-year-olds, measured during three 10 min intervals Xbox One exergames (Fruit Ninja, Kung Fu, Shape Up), categorized based on predominantly either upper-, whole-, or lower-limb movement.<br>EE was measured by COSMED K4b^2 IC, at rest and then during each activity. We calculated AEE as $EE_{activity}$ − $EE_{rest.}$	IC	REE<br>TEE<br>AEE
Ishikawa-Takata[65]	2013	Comparison of physical activity energy expenditure in Japanese adolescents assessed by EW4800P triaxial accelerometry and the doubly labelled water method	To compare the accuracy of triaxial accelerometry and DLW method for measuring physical activity.	Prospective, cross-sectional	Healthy 12–15-year-old Japanese adolescents. TEE was measured by DLW over 7 days. RMR was measured after an overnight fast after 30 min of rest that morning. AEE was calculated as 0.9TEE − RMR.	DLW<br>IC<br>C (AEE = 0.9TEE − RMR)	TEE<br>RMR<br>AEE
Jhang[66]	2020	Energy Expenditure in Mechanically Ventilated Korean Children: Single-Center Evaluation of a New Estimation Equation	To evaluate current methods for EE prediction, in comparison with IC, and developed a new EE estimation equation.	Retrospective	Critically ill, mechanically ventilated, Korean adolescents aged 17.38 ± (0.5–205.07) months (mean ± IQR). TEE was measured using CARESCAPE Monitor B650 IC.	IC	TEE
Jiang[67]	1998	Energy expenditure of Chinese infants in Guangdong Province, south China, determined with use of the doubly labeled water method	To measure EE.	Prospective, cross-sectional	Healthy 4- or 6-month-old infants. TEE was measured by DLW over 8 days.	DLW	TEE

Jindal[68]	2021	The relationship of sleep duration and quality to energy expenditure and physical activity in children	To investigate the relationship between sleep duration, quality, and timing in children, to EE and physical activity (PA).	Prospective, cross-sectional	Healthy children and adolescents aged 5–18 years (split into 5–11 years and 12–18 years).<br><br>TEE was measured by DLW over 7 days. BMR was measured for 30 min after an overnight stay in a whole room calorimeter (IC) after a 12 h fast. AEE was calculated as TEE − BMR − 0.1TEE during activities of free-living.	DLW<br>IC<br>C (AEE = TEE − BMR − 0.1TEE)	TEE<br>BMR<br>AEE
Johnson [69]	1996	Comparison of multiple-pass 24-h recall estimates of energy intake with total energy expenditure determined by the doubly labeled water method in young children	To determine the accuracy of the multiple-pass 24 h recall method for estimating energy intake in young children by comparing it with the DLW method.	Prospective, cross-sectional	Healthy white children aged 4–7 years.<br><br>TEE was measured using DLW over 14 days.	DLW	TEE
Johnson [70]	1998	Physical activity related energy expenditure in children by doubly labeled water as compared with the Caltrac accelerometer	To determine whether the caloric accelerometer was a meaningful predictor of AEE in comparison to DLW.	Prospective, cross-sectional	Healthy free-living pre-adolescent children 8.3 ± 2.0 years old (mean ±SD), made of 22 Caucasian and 9 Mohawk individuals in upstate New York and Burlington Vermont, respectively, measured over 14–16 days.<br><br>TEE was measured by DLW over 13–16 days under free-living conditions. Postprandial RMR was measured by a portable Delta-Trac Metabolic Monitor IC, one hour after a standard breakfast while lying in bed. AEE was calculated as TDEE − postprandial RMR.	DLW<br>IC<br>C (AEE = TDEE − REE)	TEE<br>RMR<br>AEE
Joosten[71]	1999	Indirect calorimetry in mechanically ventilated infants and children: accuracy of total daily energy expenditure with 2 h measurements	To determine how accurately TEE can be estimated from measurement periods of less than 24 h.	Prospective, cross-sectional	Critically ill, mechanically ventilated infants, and children aged 3 weeks–13 years. We excluded patients with chronic illness.<br>TEE was measured by the Deltatrac MBM-100 and Deltrac II MBM-200 ICs for 12 subsequent periods of 2 h.	IC	TEE
Kaskoun[72]	1994	Comparison of energy intake by semiquantitative food-frequency questionnaire with total energy expenditure by the doubly labeled water method in young children	To assess the validity of a semiquantitative food-frequency questionnaire to estimate energy intake in young children by comparison with TEE.	Interventional	Healthy Mohawk Native American and white children aged 4.2–6.9 years in Hogansburg, New York and Burlington, Vermont, respectively.<br><br>TEE was measured by DLW over 14 days.	DLW	TEE
Kim[73]	2018	Validation of Dietary Reference Intakes for predicting energy requirements in elementary school-age children	To assess the validity of Dietary Reference Intakes (DRIs) for predicting the energy requirements of elementary school-aged children.	Prospective, cross-sectional	Healthy normal-weight adolescents aged 9–11 years in Korea. Subjects are not involved in extreme physical activity and live in the study province 2 weeks before and during the study.<br><br>TEE was measured by DLW over 8 days. RMR was measured using Douglas Bag IC.	DLW<br>Douglas Bag IC	TEE<br>RMR
Komura [74]	2017	Total energy expenditure of 10- to 12-<br>year-old Japanese children measured using the doubly labeled water method	To measure TEE, to compare measured versus various TEE estimation formulas, and to develop a new TEE estimation equation using body composition and pedometers.	Prospective, cross-sectional	Healthy 10–12-year-old Japanese adolescents<br>TEE was measured by DLW over 7 days.<br>RMR was measured by IC	DLW<br>IC<br>C (AEE = 0.9TEE − RMR)	TEE<br>RMR<br>AEE

Krishnaveni[75]	2009	Relationship between physical activity measured using accelerometers and energy expenditure measured using doubly labelled water in Indian children	To test the relationship between physical activity measured using accelerometer (Actigraph) and TEE measured using DLW	Prospective, cross-sectional	Healthy Indian children aged 8–9 years measured over 14 days.	DLW	TEE
Lanigan [76]	2001	Validation of food diary method for assessment of dietary energy and macronutrient intake in infants and children aged 6–24 months	To compare the estimated food diary record method against weighted intake (WI) record method for assessing dietary intake, and to compare WI with metabolizable energy intake measured by DLW.	Interventional	Healthy infants and children aged 6–24 months, grouped into 6–12 months and 6–24 months.<br><br>TEE was measured by DLW over 7 days.	DLW	TEE
Lee[77]	2016	Activity Energy Expenditure in Youth:<br>Sex, Age, and Body Size Patterns		Prospective, cross-sectional	Healthy 7- to 13-year-olds, measured over 10 days, during 12 free-living activities picked from a list of 24 activities (**Sedentary activities**—sitting quietly in a chair, reading in a chair, watching TV (sitting), typing at a computer (sitting), playing a video game (sitting), **Light Activities**—very slow walking, loading/unloading boxes, playing catch, **Moderate to vigorous physical activities**—passing a basketball, sweeping, walking at a casual pace, brisk walking, hang weight exercises, basketball shooting, walking including stair climbing, stational cycling at moderate pace, dribbling a basketball, soccer dribbling at a slow jogging pace, light calisthenics, jogging at a slow pace, jogging as a fast pace, one-one-one basketball, stair climbing/stepping routine, stationary cycling at vigorous pace) for 5 min each with a 1 min resting interval. We designated all sedentary activities as REE, and used the lowest of the five (reading in a chair) as our "REE" in AEE calculations. We calculated AEE as AEE = $EE_{activity} - EE_{Reading\ in\ a\ chair}$.	IC<br>C (AEE = $EE_{activity} - EE_{Reading\ in\ a\ chair}$)	TEE<br>REE<br>AEE
Lindquist[78]	2012	Use of Tape-Recorded Food Records in Assessing Children's Dietary Intake	Explored the use of self-report by tape recorders to document children's dietary intake immediately upon consumption, and compared this method with the traditional, interviewer-guided recall technique.	Interventional	Healthy black and white children aged 6.5 to 11.6 years in Alabama.<br><br>TEE was measured by DLW over 14 days.	DLW	TEE
Livingstone[79]	1992	Validation of estimates of energy intake by weighed dietary record and diet history in children and adolescents	To compare energy intake by 7-day weighed dietary records and diet histories with concurrent estimates of TEE by DLW.	Prospective, cross-sectional	Healthy individuals aged 3, 5, 7, 9, 12, 15, and 18 years. TEE was measured by DLW over 10 or 14 days depending on the age of participant.	DLW	TEE
Lopez-Alarcon [80]	2004	Ability of the actiwatch accelerometer to predict free-living energy expenditure in young children	To determine if Actiwatch accelerometer measurements are associated with TEE and body composition.	Prospective, cross-sectional	Healthy White and African American children, aged 4 to 6 years, measured over 8 days.	DLW	TEE

Lucas[81]	1987	How much energy does the breast fed infant consume and expend?	To measure energy expenditure (EE), energy intake, milk volume, energy deposition, and the energy content of breast milk.	Prospective, longitudinal	Healthy, free-living, exclusively breast-fed infants (5 boys, 7 girls) measured at 5 and then 11 weeks of age, fed a formula with an energy density of 0.28 MJ/100 mL. An RQ of 0.85 was assumed in this study.<br><br>TEE was measured by DLW over 7 days. GEE was measured as energy deposited in new tissue as protein and fat.	DLW	GEE<br>TEE
Maffeis[82]	2004	Effects of dinner composition on postprandial macronutrient oxidation in prepubertal girls	To see whether a fat-rich evening meal promoted fat oxidation and a different spontaneous food intake on the following day at breakfast than a meal with a lower fat content (20%).	Prospective, cross-sectional	Healthy prepubertal obese girls aged 8–12.5 years. Two test meals fed to subjects: a low fat (LF), 2110 KJ meal made up of pasta, tomatoes, olive oil, bread, bologna cold meat (20% fat, 68% carbs, 12% protein), and a high fat (HF), 2098 KJ meal made up of pasta, tomatoes, olive oil, bread, Parma ham (50% fat, 38% carbs, 12% protein).<br><br>REE was by IC after a 12 h fast. Subjects had breakfast at 9:30 AM, lunch at 1 PM, a small snack at 4 PM, and a test meal at 7 PM after which postprandial EE was measured by IC. One week later, the subjects repeated this 24 h test with the other test meal.		REE<br>TEF
Maffeis[83]	2001	Meal-induced thermogenesis and obesity: is a fat meal a risk factor for fat gain in children?	To test the hypothesis that a mixed meal rich in fat can elicit energy saving compared with an isocaloric and isoproteic much rich in carbohydrate.	Prospective, cross-sectional	Healthy obese (BMI 25.6 ± 0.6 kg/m^2) and non-obese (BMI 19 ± 1.6 kg/m^2) girls aged 10.1 ± 0.3 years (mean ± SD).<br>The post-absorptive REE and the TEF were measured after a low-fat (LF, 20% fat, 68% carbohydrate, 12% protein) or an isocaloric (2500 kJ or 600 Cal) and isoproteic high-fat (HF, 48% fat, 40% carbohydrate, 12% protein) meal were measured by IC.<br>Each girl repeated the test with a different, randomly assigned menu (LF or HF) 1 week after the first test.	IC	REE<br>TEF
Maffeis[84]	1995	Daily energy expenditure in free-living conditions in obese and non-obese children: comparison of doubly labelled water (2H2(18)O) method and heart-rate monitoring	To compare the heart rate monitoring with the DLW method to estimate EE.	Prospective, cross-sectional	Healthy Caucasian prepubertal obese and non-obese children aged 9.1 ± 1.5 years and 9.2 ± 0.6 years (mean ± SD), respectively.<br><br>TEE was measured by DLW over 7 days.	DLW	TEE
McGloin[85]	2002	Energy and fat intake in obese and lean children at varying risk of obesity	To compare lean children at high- and low-risk of obesity, and obese children to assess the relationship between their energy intake and fat intake and adiposity.	Prospective, cross-sectional	Healthy children aged 5–8 years divided into three groups: Obese or "OB" (BMI > 95th percentile according to British growth standards), High-Risk or "HR" (at least one biological parent with a BMI over 29.5) and Low-Risk "LR" children (two lean biological parents with BMI < 25 kg/m^2)<br><br>TEE was measured by DLW over 10 days.	DLW	TEE
Montgomery[86]	2005	Validation of energy intake by 24-h multiple pass recall: comparison with total energy expenditure in children aged 5–7 years	To validate EI by 24 h multiple pass recall (MPR) DLW.	Interventional	Healthy school-aged children aged 4.5–7 years from Glasgow.<br><br>TEE was measured by DLW over 10 days.	DLW	TEE

Nie lsen [87]	2012	Validation of energy requirement references for exclusively breast-fed infants	To measure EE using criterion methods and validate the use of TEE prediction equation and mean energy requirement references for predicting individual EE.	Prospective, longitudinal	Healthy Scottish infants, exclusively breast-fed to 6 months of age, measured at 3.5 and 6 months of age.<br><br>TEE was measured by DLW over 7 days.	DLW	TEE
Nicklas[88]	2017	Validity of the Remote Food Photography Method Against Doubly Labeled Water Among Minority Preschoolers	To determine the validity of energy intake (EI) estimations made using the remote food photography method (RFFM) compared to the DLW method.	Prospective, cross-sectional	Healthy Hispanic and African American preschool children aged 3–5-years, in a free-living environment.<br><br>TEE was measured by DLW over 7 days.	DLW	TEE
O'Connor[89]	2001	Comparison of total energy expenditure and energy intake in children aged 6–9 y	To compare measurements of EE and Energy Intake (EI)	Prospective, cross-sectional	Healthy children aged 7.4 ± 0.8 years (mean ± SD) measured over 10 days. Energy intake was measured using 3-day food records.	DLW	TEE
Ojiambo [90]	2012	Validity of hip-mounted uniaxial accelerometry with heart-rate monitoring vs. triaxial accelerometry in the assessment of free-living energy expenditure in young children: the IDEFICS Validation Study	To test the validity of hip-mounted uniaxial accelerometry with heart-rate monitoring vs triaxial accelerometer.	Interventional	Healthy children aged 4–10 years. TEE was measured by DLW over 7 days.	DLW	TEE
Ojiambo [91]	2013	Free-living physical activity and energy expenditure of rural children and adolescents in the Nandi region of Kenya	To examine the relationship between physical activity and energy demands in children and adolescents with highly active lifestyles.	Prospective, cross-sectional	Healthy rural Kenyan children and adolescents 14 ± 1 years (mean ± SD). TEE was measured by DLW over 7 days.	DLW	TEE
Park[92]	2018	Comparison of daily physical activity parameters using objective methods between overweight and normal-weight children	To determine any differences in aspects of physical activity such as EE, intensity, and activity type between normal and overweight boys in Korea.	Prospective, cross-sectional	Healthy normal and overweight children and adolescents aged 9–12 years in Korea, measured over 7 days during activities of daily living. Children were not involved in hard physical activity such as young athletes, and who lived at home.<br><br>TEE was measured by DLW, over 7 days. RMR was measured by IC in the early morning, 12 h or longer after the last meal. AEE was calculated as 0.9TEE − REE.	DLW<br>Douglas Bag IC<br>C (AEE = 0.9TEE − RMR)	TEE<br>RMR<br>AEE
Park[93]	2016	Association between daily step counts and physical activity level among Korean elementary schoolchildren	To investigate steps/day and physical activity level (PAL) in Korean elementary school children of a normal weight.	Prospective, cross-sectional	Healthy adolescents aged 9–12 years in Korea, not involved in hard physical activity such as young athletes, and who lived at home.<br>TEE was measured by DLW over 8 days.<br>RMR was measured by IC in the early morning, 12 h or longer after the last meal.	DLW<br>Douglas Bag IC	TEE<br>RMR
Perks[94]	2000	Alterations in growth and body composition during puberty. IV. Energy intake estimated by the youth-adolescent food-frequency questionnaire:	To validate energy intake estimated by the Youth-Adolescent Food-Frequency Questionnaire (YAQ) against TEE measurements using DLW.	Prospective, cross-sectional	Healthy 8.6–16.2-year-old adolescents. TEE was measured by DLW over 12 days. BMR was measured by IC for 30 min on waking after an overnight stay at the research centre.	DLW<br>IC	TEE<br>BMR

		validation by the doubly labeled water method					
Pfeiffer[95]	2006	Physical activities in adolescent girls: variability in energy expenditure	To describe variability in EE for selected physical activities.	Prospective, cross-sectional	Healthy adolescents aged 13–14 years.<br><br>EE was measured by IC for 10 activities (rest, tv viewing, playing computer game, sweeping, slow walk, brisk walk, step aerobics, bicycle riding, shooting baskets, stair climbing, and running) and during a submaximal cycle ergometer test. We counted rest, TV viewing, and playing computer games as REE measurements, and used the lowest of the three (Rest) as our “REE” during AEE calculations.<br><br>EE during activities was measured by IC. AEE was calculated as $EE_{activity}$ − REE.	IC<br>C (AEE = $EE_{activity}$ − REE)	REE<br>AEE
Prelack[96]	2017	Measures of Total Energy Expenditure and Its Components Using the Doubly Labeled Water Method in Rehabilitating Burn Children	To measure EE.	Prospective, cross-sectional	Healthy rehabilitating pediatric burns patients (children with initial burns of 20% of their body surface area or greater), aged 10.4 ± 5.5 years (mean ±SD). We classified burns patients as “healthy” as patients who were in rehabilitation, not critically ill. TEE was measured by DLW over 7 days. REE was measured on 2 days by IC.	DLW<br>IC	TEE<br>REE
Ramirez-Marrero[97]	2005	Comparison of methods to estimate physical activity and energy expenditure in African American children	To compare methods of the Tritrac-R3D accelerometer, Yamax SW-200 Digiwalked step-counter and Send Administered Physical Activity Checklist (SAPAC) to estimate physical activity and EE.	Interventional	Healthy African American Children aged 7–10 years.<br><br>TEE was measured by DLW over 7 days.	DLW	TEE
Ramuth[98]	2020	Total energy expenditure assessed by doubly labeled water technique and estimates of physical activity in Mauritian children: analysis by gender and ethnicity	To measure TEE in Mauritian children and explore gender- and ethnicity-related differences.	Prospective, cross-sectional	Healthy Mauritian children from either Indian (South Asian descent) or Creole (African/Malagasy descent), aged 7–11 years, measured over 14 days.	DLW	TEE
Reilly[99]	2006	Validation of Actigraph accelerometer estimates of total energy expenditure in young children	To assess the validity of the equations based on the Actigraph CSA/MTI accelerometer through comparison with DLW measured TEE.	Prospective, cross-sectional	Healthy preschool-aged and school-aged participants (mean (SD) 4.6 ± 1.1 years), measured for 7 and 10 days, respectively.	DLW	TEE
Reilly[100]	2001	Energy intake by multiple pass 24 h recall and total energy expenditure: a comparison in a representative sample of 3–4-year-olds	To assess the accuracy of 24 h recall by comparison with measurement by DLW.	Prospective, cross-sectional	Healthy children aged 3–4 years in Glasgow, Scotland.<br><br>DLW was measured in free-living subjects over 7 days.	DLW	TEE
Rennie[101]	2005	Association of physical activity with body-composition indexes in children aged 6–8 y at varied risk of obesity	To determine the relationship between physical activity levels and body fatness.	Prospective, cross-sectional	Healthy children aged 6–8 years at high-risk (≥ one obese biological parent) and low-risk for obesity (two non-obese biological parents), measured over 7 days.	DLW	TEE

Roemmich[102]	1998	Gender differences in leptin levels during puberty are related to the subcutaneous fat depot and sex steroids	To examine the relationships among gender, sexual maturation, four-compartment model estimates of body composition, body fat distribution, aerobic fitness, BMR and TEE, and overnight GH release in ultrasensitive chemiluminescence assay in healthy boys.	Prospective, cross-sectional	Healthy pubertal and prepubertal individuals (3 obese), assessed on waking.<br><br>BMR was measured after an overnight fast by IC for 30 min IC (Deltatrac). TEE was measured by DLW over 12 days.	DLW (Deltatrac) IC	TEE BMR
Roemmich[103]	2000	Pubertal alterations in growth and body composition. V. Energy expenditure, adiposity, and fat distribution	To determine whether AEE or physical activity [7-day physical activity recall (PAR)] was more related to adiposity and the validity of PAR estimated total energy expenditure in prepubertal and pubertal boys and girls.	Prospective, cross-sectional	Prepubertal and pubertal adolescents enrolled in a longitudinal growth study.<br><br>TEE was measured by DLW over a 12-day period.<br>REE was measured by IC.	DLW<br>IC<br>C (AEE = TEE − RMR)	TEE<br>REE<br>AEE
Rush[104]	2003	Body composition and physical activity in New Zealand Maori, Pacific and European children aged 5–14 years	To measure body fatness and EE in Maori, Pacific, and European children to determine ethnic differences in these components.	Prospective, cross-sectional	Healthy New Zealand Māori, Pacific, and European children aged 5–14 years. TEE was measured by DLW over a 10-day period. REE was measured by IC under postprandial conditions (1–3 h after their usual lunch) after school. AEE was calculated as TEE − postprandial RMR.	DLW<br>IC<br>C (AEE = TEE − postprandial RMR)	TEE<br>REE<br>AEE
Salas-Salvados[105]	1993	Influence of adiposity on the thermic effect of food and exercise in lean and obese adolescents	To determine the effect of adiposity on adolescent EE compartments, BMR, together with TEF at rest and during post-exercise recovery.	Prospective, cross-sectional	Healthy lean and obese 15-year-old males at the same stage of pubertal development and with equivalent FFM, measured after an overnight fast, a test meal (a 795 ± 113 kcal and 876 ± 127 kcal [30% protein, 32% fat, 38% carbohydrate] meal in lean and obese groups, respectively), and moderate exercise after a further serving of the test meal (ergometer at 70% of PWC170 [the intensity of physical activity required to reach a cardiac frequency of 170 beats/min, based on previous tests]). Postprandial EE (PEE), postprandial/post-exercise EE (PPEE), postprandial thermogenesis (PT), and postprandial/post-exercise thermogenesis (PPT) were calculated from measured values.<br><br>EE was measured by IC (open-circuit SensorMedics EE Unit 2900). REE was measured the morning after consuming a 600 kcal liquid diet.<br><br>We calculated AEE as<br>PPEE − PEE, to isolate the effects of activity.	IC<br>C (AEE = PPEE − PEE)	REE<br>AEE
Salazar[106]	2015	Energy Expenditure and Intake Comparisons in Chilean Children 4–5 Years Attending Day-Care Centres	To measure EE and compare to RQ values used in the literature.	Prospective, cross-sectional	Healthy preschool children 4 to 5 years of age, measured over 14 days at either a high dose (0.2 g/kg deuterium oxide (99%) and 2.0 g/kg 10% H218O)) or<br>low dose (0.08 g/kg deuterium oxide (99%) and 1.55 g/kg 10% H218O).	DLW	TEE
Salbe[107]	1997	Total energy expenditure and the level of physical activity correlate with plasma leptin concentrations in five-year-old children	To assess the relationship between fasting plasma leptin concentrations and energy expenditure in Pima Indian Children.	Interventional	Healthy 5-year-old Pima Indian Children, after an overnight fast, one week apart.<br><br>RMR was measured while fasted, for 20 min using the DeltaTrac Metabolic Monitor, while resting on a bed.	DLW<br>IC<br>(DeltaTrac Metabolic Monitor)	TEE<br>RMR

Salbe[108]	2002	Assessing risk factors for obesity between childhood and adolescence: II. Energy metabolism and physical activity	To assess the effect of EE, as well as substrate oxidation (Respiratory Quotient/RQ) on the development of obesity in a large cohort of Native American children with a high propensity for obesity.	Prospective, cross-sectional	Healthy Pima Indian children measured at 5 years, and then 10 years old.<br><br>TEE was measured by DLW over 7 days. REE was measured by DeltaTrac Metabolic Monitor (IC) for 20 min, after lying on a bed for 10 min. AEE was calculated as RMR + 0.1TEE during activities of free-living, where 0.1TEE represents an estimate of TEF.	DLW<br>IC<br>C (RMR + 0.1TEE)	TEE<br>RMR<br>AEE
Sijtsma[109]	2013	Validation of the TracmorD triaxial accelerometer to assess physical activity in preschool children	To assess validity evidence of TracmorD to determine PAEE in 3-year old children.	Prospective, cross-sectional	Healthy children aged 3.4 ± 0.3 years (mean ±SD).<br><br>TEE was measured by DLW over 7 days. SMR was measured by IC. AEE was calculated as TEE − SMR.	DLW<br>IC (Deltatrac)<br>C (AEE = TEE − SMR)	TEE<br>SMR<br>AEE
Singh[110]	2009	Comparison of self-reported, measured, metabolizable energy intake with total energy expenditure in overweight teens	To assess the energy reporting bias of diet records against the reference TEE and to compare the methods of determining EE through metabolizable energy intake (MEI) and TEE.	Interventional	Healthy adolescents aged 12–15 years, measured over 3-week metabolic balance sessions, separated by a 3-week washout period to study the effects of calcium intake on predictors of fat balance.<br><br>TEE was measured by DLW over 14 days. REE was measured with IC while fasted, while rested in a recumbent position for ~35 min.	DLW<br>IC	TEE<br>REE
Sjoberg[111]	2003	Energy intake in Swedish adolescents: validation of diet history with doubly labelled water		Prospective, cross-sectional	Healthy adolescents aged 15.7± 0.4 years old (mean ± SD). TEE was measured by DLW over 14 days.	DLW	TEE
Slinde[112]	2003	Minnesota leisure time activity questionnaire and doubly labeled water in adolescents	To validate the energy expenditure estimated from the Minnesota Leisure Time Physical Activity Questionnaire (MLTPAQ) with DLW measurements and to present and examine the validity of an extended version of the MLTPAQ with additional questions about inactivity during leisure time.	Prospective, cross-sectional	Healthy adolescents aged 15–17-years in Sweden. TEE was measured by DLW over 14 days. BMR was measured by IC.	DLW<br>IC	TEE<br>BMR<br>AEE
Spadano[113]	2003	Energy cost of physical activities in 12-y-old girls: MET values and the influence of body weight	To provide MET data on five specific activities in 12-year-old girls and to test the hypothesis that measured MET values are independent of body weight.	Prospective, cross-sectional	Healthy, premenarcheal, 12-year-old girls with a triceps skinfold thickness less than the 85th percentile for age and sex, measured either on evening of admittance to research centre or the next morning approx. 2 h after breakfast,<br>during five activities (sitting, standing, walking on a flat treadmill at 3.2 and 4.8 km/h, and walking on a treadmill at a 10% incline at 4.8 km/h). IC measurements were collected over 4 min for sitting and standing, 3 min for walking 3.2 km/h and 2 min each for walking on a flat surface and at a 10% incline at 4.8 km/h.	IC	TEE<br>AEE
Sun[114]	1998	Total, resting, and activity-related energy expenditures are similar in Caucasian and African-American children	To measure EE.	Prospective, cross-sectional	Healthy prepubertal Caucasian and African American children aged 8.3 ± 1.4 years (mean ± SD) from Alabama.<br><br>TEE was measured by DLW over 14 days under free-living conditions.<br>REE was measured after an overnight fast using IC.	DLW<br>IC<br>C (AEE = 0.9(TEE) − REE)	TEE<br>REE<br>AEE

					AEE was calculated as 0.9(TEE) – REE.		
Tennefors[115]	2003	Total energy expenditure and physical activity level in healthy young Swedish children 9 or 14 months of age	To measure EE, to compare TEE with recommendations for energy intake, and to define body composition in relation to EE.	Prospective, cross-sectional	Healthy Swedish children aged 9 and 14 months, measured over 11 days.	DLW	TEE
Treuth[116]	1998	Energy expenditure and physical fitness in overweight vs non-overweight prepubertal girls	To determine whether overweight children have lower PAEE and fitness levels than non-overweight children.	Prospective, cross-sectional	Healthy overweight (>95th percentile weight-for-height) and non-overweight (10–90th percentile weight-for-height) girls aged 7–10 years in Alabama. Subjects were fed meals with approximately 50% carbohydrate, 30% fat, and 20% protein, at around 8:00,12:00: and 17:00 h with snacks at 10:00 and 14:00 h. Non-overweight children were fed ad libitum (350 kJ/kg body weight), and overweight children were fed based on weight (188 kJ/kg body weight).<br>TEE was measured by DLW over 14 days. BMR was measured after an overnight fast by IC (Deltatrac system).<br>AEE was calculated as TEE − (BMR + 0.1TEE).	DLW<br>IC (Deltatrac system)<br>C (AEE = TEE − (BMR + 0.1TEE))	TEE<br>SMR<br>SMR<br>AEE
Urlacher[117]	2019	Constraint and trade-offs regulate energy expenditure during childhood	To test for constraints and trade-offs in childhood EE using DLW measures or TEE and respirometry measures of REE.	Prospective, cross-sectional	Healthy forager-horticulturalist Shuar children aged 5–12 years, from Amazonian Ecuador. TEE was measured by DLW over 11 days. REE was measured on days 0, 7, and 14 using Cosmed IC. AEE was calculated as TEE − (REE +0.1TEE) during activities of free-living.	DLW<br>IC (Cosmed)<br>C (AEE = TEE − (REE +0.1TEE))	TEE<br>REE<br>AEE
Urlacher[118]	2021	Childhood Daily Energy Expenditure Does Not Decrease with Market Integration and Is Not Related to Adiposity in Amazonia	To measure EE to investigate the changes that underlie childhood overweight and obesity (OW/OB) and the nutrition/epidemiological transition.	Prospective, cross-sectional	Healthy forager-horticulturalist Shuar children and their "peri-urban" counterparts aged 4–12 years, in Amazonian Ecuador, measured over 14 days.<br>TEE was measured by DLW. REE was measured by Cosmed IC. AEE was calculated as TEE − (REE + 0.1TEE) during activities of free-living.	DLW<br>IC (Cosmed)<br>C (AEE = TEE − (REE +0.1TEE))	TEE<br>REE<br>AEE
Valencia [119]	1995	Energetic consequences of mild Giardia intestinalis infestation in Mexican children	To explore the effects of mild infestation with Giardia on energy intake and EE.	Interventional	Healthy Mexican 6–10-year-olds with a mild Giardia infestation. TEE was measured by DLW over 7 days. BMR was measured by IC using a Deltatrac ventilated hood system while the child watched TV. EE was measured both during infestation and after treatment.	DLW<br>IC	TEE<br>BMR
Van der Kuip[120]	2007	Physical activity as a determinant of total energy expenditure in critically ill children	To investigate EE and relation with physical activity during critical illness and initial recovery.	Prospective, cross-sectional	Critically ill infants, children, and adolescents aged 0–16 years, with sepsis or following surgery, with 11 patients receiving enteral feedings exclusively and 9 patients receiving parenteral nutrition provided as all-in-one mixtures with an energy density between 2.3 and 4.7 MJL^{-1} (glucose: 73%, protein: 12%, fat 15%).<br>TEE was measured by DLW over approximately 7 days. REE was measured by Deltatrac II MBM-200 IC.	DLW<br>IC (Deltatrac II MBM-200)	TEE<br>REE
Vasquez [121]	2006	Energy balance and physical activity in obese children attending day-care centres	To evaluate energy balance and physical activity in preschool children attending daycare centres.	Prospective, cross-sectional	Healthy obese 3–5-year-old children attending daycare in Chile.<br>TEE was measured by DLW after breaking a 2 h fast with 100 mL of milk without any solid incorporated.	DLW	TEE

Vazquez Martinez[122]	2004	Predicted versus measured energy expenditure by continuous, online indirect calorimetry in ventilated, critically ill children during the early postinjury period	To compare the EE predicted by anthropometric equations, with that measured by continuous on-line IC.	Prospective, cross-sectional	Critically ill ventilated children during the first 6 h after injury, aged 4.21 ± 3.67 years (mean ± SD), at a pediatric intensive care unit in Spain.<br><br>IC was used to measure EE for 24 h continuously.	IC	TEE
Waling[123]	2009	Energy intake of Swedish overweight and obese children is underestimated using a diet history interview	To validate reported EI from a Diet History Interview (DHI) in children classified as overweight or obese by comparing the reported EI to TEE measured by two objective measures.	Retrospective interventional	Healthy overweight (BMI of 25–29.9 kg/m^2) and obese (BMI of ≥30 kg/m^2) Swedish children aged 10.5 ± 1.1 years (mean ± SD) recruited to a 2 y interventional study on food and physical activity habits.<br><br>TEE was measured by DLW over 14 days.	DLW	TEE
Wells[124]	1996	Total energy expenditure and body composition in early infancy	To measure the relationship between body composition and EE.	Prospective, cross-sectional	Healthy full-term infants at 12 weeks old measured over 8 days.	DLW	TEE
Wells[125]	1995	Energy Cost of Physical Activity in Twelve Week Old Infants	To measure EE.	Prospective, cross-sectional	Healthy, free-living, breast-fed, and formula-fed infants at 12 weeks old measured over 7 days.	DLW<br>C (AEE = TDEE − SMR)	TEE<br>AEE
Wells[126]	1998	Estimation of the energy cost of physical activity in infancy	To estimate PAEE.	Prospective, longitudinal	Healthy, free-living infants measured at 6 weeks, 12 weeks, 6 months, 9 months, and 12 months.<br>TEE was measured by DLW over 8 days.	DLW	TEE
Wells[127]	1997	Free-living energy expenditure and behaviour in late infancy	To study the relationship between EE and behaviour.	Prospective, cross-sectional	Healthy, full-term infants aged 9 and 12 months.<br><br>TEE was measured by DLW over 7 days.	DLW	TEE
White[128]	1999	Energy expenditure measurements in ventilated critically ill children: within- and between-day variability	To determine if a 30 min EE measurement (EEM) is representative of a 24 h EEM, to determine if there is any diurnal variation during the 24 h period of EEM (within-day variation), and to determine if there is a clinically significant between day variation of EEMs.	Prospective, cross-sectional	Critically ill ventilated children in an ICU aged 0.1 to 13 years.<br><br>TEE was measured by the Deltatrac IC metabolic cart IC in 30 min periods over 24 h.	IC (Deltatrac II Metabolic Cart)	TEE
Wong[129]	1994	Energy expenditure of female adolescents	To measure EE and define energy requirements.	Prospective, cross-sectional	Healthy female Caucasian adolescents aged 10.3–16 years.<br><br>TEE was measured by DLW over 10 days.	DLW	TEE
Wren[130]	1997	Body composition, resting metabolic rate, and energy requirements of short- and normal-stature, low-income Guatemalan children	To measure EE.	Prospective, cross-sectional	Healthy short-stature (height-for-age ≤ −1.5 SD) and normal-stature (height-for-age > −1.5 SD) Guatemalan children aged 4–6 years, over a 4-week study period.<br><br>RMR was measured by portable Deltatrac II Metabolic Monitor IC, under fasting conditions, twice during the study period.<br>TEE was measured by DLW under free-living conditions over 7d.<br>AEE was calculated as TEE − RMR − TEF. Because RMR was measured in fasted conditions, TEF and energy from physical exertion were accounted for in AEE calculations.	DLW<br>IC<br>C (AEE = TEE − RMR − TEF)	TEE<br>RMR<br>AEE

Yamada [131]	2020	Total Energy Expenditure, Body Composition, Physical Activity, and Step Count in Japanese Preschool Children: A Study Based on Doubly Labeled Water	To measure TEE and determine if it can be estimated by Fat-Free Mass (FFM) and step count.	Prospective, cross-sectional	Healthy Japanese preschool children aged 4–6 years, measured over 7 days.	DLW	TEE
Yamada [132]	2021	Association between Water and Energy Requirements with Physical Activity and Fat-Free Mass in Preschool Children in Japan	To examine the association of water turnover and TEE with physical activity and body composition in Japanese children in preschool.	Prospective, cross-sectional	Healthy Japanese preschool children aged 3–6 years, measured over 7 days.	DLW	TEE
Zinkel[133]	2013	Comparison of total energy expenditure between school and summer months	To analyze TEE data from school-age African American and Caucasian youth in or near District of Columbia who were at risk of obesity.	Prospective, cross-sectional	Healthy, school-aged sedentary African American and Caucasian youth aged 10 ± 2 years (mean ± SD), at risk of obesity (BMI ≥ 85th percentile or had overweight parents). TEE was measured by DLW over 7 days either between September and June or June and August.	DLW	TEE

References

1. Abbott, R.A.; Davies, P.S.W. Habitual physical activity and physical activity intensity: Their relation to body composition in 5.0-10.5-y-old children. *Eur J Clin Nutr* **2004**, *58*, 285-291, doi:http://dx.doi.org/10.1038/sj.ejcn.1601780.
2. Arvidsson, D.; Slinde, F.; Hulthen, L. Physical activity questionnaire for adolescents validated against doubly labelled water. *Eur J Clin Nutr* **2005**, *59*, 376-383.
3. Arvidsson, D.; Slinde, F.; Hulthen, L. Free-living energy expenditure in children using multi-sensor activity monitors. *Clinical nutrition* **2009**, *28*, 305-312, doi:http://dx.doi.org/10.1016/j.clnu.2009.03.006.
4. Backlund, C.; Sundelin, G.; Larsson, C. Validity of armband measuring energy expenditure in overweight and obese children. *Medicine and Science in Sports and Exercise* **2010**, *42*, 1154-1161, doi:http://dx.doi.org/10.1249/MSS.0b013e3181c84091.
5. Ball, E.J.; O'Connor, J.; Abbott, R.; Steinbeck, K.S.; Davies, P.S.; Wishart, C.; Gaskin, K.J.; Baur, L.A. Total energy expenditure, body fatness, and physical activity in children aged 6-9 y. *Am J Clin Nutr* **2001**, *74*, 524-528.
6. Bandini, L.G.; Schoeller, D.A.; Dietz, W.H. Energy expenditure in obese and nonobese adolescents. *Pediatric research* **1990**, *27*, 198-203.
7. Bandini, L.G.; Must, A.; Spadano, J.L.; Dietz, W.H. Relation of body composition, parental overweight, pubertal stage, and race-ethnicity to energy expenditure among premenarcheal girls. *Am J Clin Nutr* **2002**, *76*, 1040-1047.
8. Bandini, L.G.; Schoeller, D.A.; Edwards, J.; Young, V.R.; Oh, S.H.; Dietz, W.H. Energy expenditure during carbohydrate overfeeding in obese and nonobese adolescents. *Am J Physiol* **1989**, *256*, E357-367.
9. Bell, K.L.; Davies, P.S. Energy expenditure and physical activity of ambulatory children with cerebral palsy and of typically developing children. *Am J Clin Nutr* **2010**, *92*, 313-319, doi:http://dx.doi.org/10.3945/ajcn.2010.29388.
10. Bitar, A.; Fellmann, N.; Vernet, J.; Coudert, J.; Vermorel, M. Variations and determinants of energy expenditure as measured by whole-body indirect calorimetry during puberty and adolescence. *Am J Clin Nutr* **1999**, *69*, 1209-1216.
11. Bornhorst, C.; Bel-Serrat, S.; Pigeot, I.; Huybrechts, I.; Ottavaere, C.; Sioen, I.; De Henauw, S.; Mouratidou, T.; Mesana, M.I.; Westerterp, K.; et al. Validity of 24-h recalls in (pre-)school aged children: Comparison of proxy-reported energy intakes with measured energy expenditure. *Clinical nutrition* **2014**, *33*, 79-84, doi:http://dx.doi.org/10.1016/j.clnu.2013.03.018.
12. Brage, S.; Lindsay, T.; Venables, M.; Wijndaele, K.; Westgate, K.; Collins, D.; Roberts, C.; Bluck, L.; Wareham, N.; Page, P. Descriptive epidemiology of energy expenditure in the UK: findings from the National Diet and Nutrition Survey 2008-15. *Int J Epidemiol* **2020**, *49*, 1007-1021, doi:10.1093/ije/dyaa005.
13. Brandes, M.; Steenbock, B.; Wirsik, N. Energy Cost of Common Physical Activities in Preschoolers. *J Phys Act Health* **2018**, *15*, 233-238, doi:10.1123/jpah.2017-0348.

14. Bratteby, L.E.; Sandhagen, B.; Fan, H.; Samuelson, G. A 7-day activity diary for assessment of daily energy expenditure validated by the doubly labelled water method in adolescents. *Eur J Clin Nutr* **1997**, *51*, 585-591.
15. Bratteby, L.E.; Sandhagen, B.; Fan, H.; Enghardt, H.; Samuelson, G. Total energy expenditure and physical activity as assessed by the doubly labeled water method in Swedish adolescents in whom energy intake was underestimated by 7-d diet records. *Am J Clin Nutr* **1998**, *67*, 905-911.
16. Burrows, T.L.; Truby, H.; Morgan, P.J.; Callister, R.; Davies, P.S.; Collins, C.E. A comparison and validation of child versus parent reporting of children's energy intake using food frequency questionnaires versus food records: who's an accurate reporter? *Clinical nutrition* **2013**, *32*, 613-618, doi:http://dx.doi.org/10.1016/j.clnu.2012.11.006.
17. Butte, N.F.; Wong, W.W.; Ferlic, L.; Smith, E.O.; Klein, P.D.; Garza, C. Energy expenditure and deposition of breast-fed and formula-fed infants during early infancy.[Erratum appears in Pediatr Res 1991 May;29(5):454]. *Pediatric research* **1990**, *28*, 631-640.
18. Butte, N.F.; Wong, W.W.; Hopkinson, J.M.; Heinz, C.J.; Mehta, N.R.; O'Brian Smith, E. Energy requirements derived from total energy expenditure and energy deposition during the first 2 y of life. *Am J Clin Nutr* **2000**, *72*, 1558-1569.
19. Butte, N.F.; Wong, W.W.; Lee, J.S.; Adolph, A.L.; Puyau, M.R.; Zakeri, I.F. Prediction of energy expenditure and physical activity in preschoolers. *Med Sci Sports Exerc* **2014**, *46*, 1216-1226, doi:http://dx.doi.org/10.1249/MSS.0000000000000209.
20. Butte, N.F.; Puyau, M.R.; Wilson, T.A.; Liu, Y.; Wong, W.W.; Adolph, A.L.; Zakeri, I.F. Role of physical activity and sleep duration in growth and body composition of preschool-aged children. *Obesity (Silver Spring)* **2016**, *24*, 1328-1335, doi:10.1002/oby.21489.
21. Butte, N.E.; Wong, W.W.; Adolph, A.L.; Puyau, M.R.; Vohra, F.A.; Zakeri, I.F. Validation of Cross-Sectional Time Series and Multivariate Adaptive Regression Splines Models for the Prediction of Energy Expenditure in Children and Adolescents Using Doubly Labeled Water. *J Nutr* **2010**, *140*, 1516-1523.
22. Byun, W.; Barry, A.; Lee, J.M. Energy Expenditure of Daily Living Activities in 3- to 6-Year-Old Children. *J Phys Act Health* **2016**, *13*, S3-S6, doi:http://dx.doi.org/10.1123/jpah.2015-0715.
23. Calabro, M.A.; Stewart, J.M.; Welk, G.J. Validation of pattern-recognition monitors in children using doubly labeled water. *Med Sci Sports Exerc* **2013**, *45*, 1313-1322, doi:http://dx.doi.org/10.1249/MSS.0b013e31828579c3.
24. Campbell, N.; Prapavessis, H.; Gray, C.; McGowan, E.; Rush, E.; Maddison, R. The Actiheart in adolescents: a doubly labelled water validation. *Pediatr Exerc Sci* **2012**, *24*, 589-602.
25. Carter, J.; Wilkinson, D.; Blacker, S.; Rayson, M.; Bilzon, J.; Izard, R.; Coward, A.; Wright, A.; Nevill, A.; Rennie, K.; et al. An investigation of a novel three-dimensional activity monitor to predict free-living energy expenditure. *J Sports Sci* **2008**, *26*, 553-561, doi:http://dx.doi.org/10.1080/02640410701708979.
26. Collins, C.E.; Burrows, T.L.; Truby, H.; Morgan, P.J.; Wright, I.M.; Davies, P.S.; Callister, R. Comparison of energy intake in toddlers assessed by food frequency questionnaire and total energy expenditure measured by the doubly labeled water method. *J Acad Nutr Diet* **2013**, *113*, 459-463, doi:http://dx.doi.org/10.1016/j.jand.2012.09.021.
27. Corder, K.; van Sluijs, E.M.; Wright, A.; Whincup, P.; Wareham, N.J.; Ekelund, U. Is it possible to assess free-living physical activity and energy expenditure in young people by self-report? *Am J Clin Nutr* **2009**, *89*, 862-870, doi:http://dx.doi.org/10.3945/ajcn.2008.26739.
28. Corder, K.; Brage, S.; Wright, A.; Ramachandran, A.; Snehalatha, C.; Yamuna, A.; Wareham, N.J.; Ekelund, U. Physical activity energy expenditure of adolescents in India. *Obesity (Silver Spring)* **2010**, *18*, 2212-2219, doi:http://dx.doi.org/10.1038/oby.2010.4.
29. Dabare, P.; Wickramasinghe, P.; Waidyatilaka, I.; Devi, S.; Kurpad, A.V.; Samaranayake, D.; de Lanerolle-Dias, M.; Wickremasinghe, R.; Hills, A.P.; Lanerolle, P. Prediction Equation for Physical Activity Energy Expenditure in 11-13-Year-Old Sri Lankan Children. *Nutrients* **2023**, *15*, doi:10.3390/nu15040906.
30. Davidsson, L.; Al-Ghanim, J.; Al-Ati, T.; Al-Hamad, N.; Al-Mutairi, A.; Al-Olayan, L.; Preston, T. Total energy expenditure in obese kuwaiti primary school children assessed by the doubly-labeled water technique. *International Journal of Environmental Research and Public Health* **2016**, *13 (10) (no pagination)*, doi:http://dx.doi.org/10.3390/ijerph13101007.
31. Davies, P.S.W.; Ewing, G.; Lucas, A. Energy expenditure in early infancy. *Br J Nutr* **1989**, *62*, 621-629, doi:http://dx.doi.org/10.1079/BJN19890062.
32. Davies, P.S.; Wells, J.C.; Hinds, A.; Day, J.M.; Laidlaw, A. Total energy expenditure in 9 month and 12 month infants. *Eur J Clin Nutr* **1997**, *51*, 249-252.
33. Davies, P.S.W.; Day, J.M.E.; Lucas, A. Energy expenditure in early infancy and later body fatness. *Int J Obes (Lond)* **1991**, *15*, 727-731.
34. Davies, P.S.; Coward, W.A.; Gregory, J.; White, A.; Mills, A. Total energy expenditure and energy intake in the pre-school child: a comparison. *Br J Nutr* **1994**, *72*, 13-20.

35. Davies, P.S.; Gregory, J.; White, A. Physical activity and body fatness in pre-school children. *Int J Obes Relat Metab Disord* **1995**, *19*, 6-10.
36. Davies, P.S.; Gregory, J.; White, A. Energy expenditure in children aged 1.5 to 4.5 years: a comparison with current recommendations for energy intake. *Eur J Clin Nutr* **1995**, *49*, 360-364.
37. de Bruin, N.C.; Degenhart, H.J.; Gal, S.; Westerterp, K.R.; Stijnen, T.; Visser, H.K.A. Energy utilization and growth in breast-fed and formula-fed infants measured prospectively during the first year of life. *Am J Clin Nutr* **1998**, *67*, 885-896.
38. DeLany, J.P.; Bray, G.A.; Harsha, D.W.; Volaufova, J. Energy expenditure in preadolescent African American and white boys and girls: the Baton Rouge Children's Study. *Am J Clin Nutr* **2002**, *75*, 705-713.
39. DeLany, J.P.; Bray, G.A.; Harsha, D.W.; Volaufova, J. Energy expenditure in African American and white boys and girls in a 2-y follow-up of the Baton Rouge Children's Study. *Am J Clin Nutr* **2004**, *79*, 268-273.
40. Delisle Nystrom, C.; Henriksson, H.; Alexandrou, C.; Bergstrom, A.; Bonn, S.; Balter, K.; Lof, M. Validation of an Online Food Frequency Questionnaire against Doubly Labelled Water and 24 h Dietary Recalls in Pre-School Children. *Nutrients* **2017**, *9*, 13, doi:https://dx.doi.org/10.3390/nu9010066.
41. Dugas, L.R.; Ebersole, K.; Schoeller, D.; Yanovski, J.A.; Barquera, S.; Rivera, J.; Durazo-Arzivu, R.; Luke, A. Very low levels of energy expenditure among pre-adolescent Mexican-American girls. *Int J Pediatr Obes* **2008**, *3*, 123-126, doi:http://dx.doi.org/10.1080/17477160801902248.
42. Dutman, A.E.; Stafleu, A.; Kruizinga, A.; Brants, H.A.; Westerterp, K.R.; Kistemaker, C.; Meuling, W.J.; Goldbohm, R.A. Validation of an FFQ and options for data processing using the doubly labelled water method in children. *Public health nutrition* **2011**, *14*, 410-417, doi:http://dx.doi.org/10.1017/S1368980010002119.
43. Ekelund, U.; Sjostro, M.; Yngve, A.; Poortvliet, E.; Nilsson, A.; Froberg, K.; Wedderkopp, N.; Westerterp, K. Physical activity assessed by activity monitor and doubly labeled water in children. *Medicine and Science in Sports and Exercise* **2001**, *33*, 275-281.
44. Ekelund, U.; Aman, J.; Yngve, A.; Renman, C.; Westerterp, K.; Sjostrom, M. Physical activity but not energy expenditure is reduced in obese adolescents: a case-control study. *Am J Clin Nutr* **2002**, *76*, 935-941.
45. Ekelund, U.; Yngve, A.; Brage, S.; Westerterp, K.; Sjostrom, M. Body movement and physical activity energy expenditure in children and adolescents: how to adjust for differences in body size and age. *Am J Clin Nutr* **2004**, *79*, 851-856.
46. Eliakim, A.; Scheett, T.; Allmendinger, N.; Brasel, J.A.; Cooper, D.M. Training, muscle volume, and energy expenditure in nonobese American girls. *Journal of applied physiology* **2001**, *90*, 35-44.
47. Elliott, S.A.; Baxter, K.A.; Davies, P.S.; Truby, H. Accuracy of self-reported physical activity levels in obese adolescents. *J Nutr Metab* **2014**, *2014*, 808659, doi:10.1155/2014/808659.
48. Fisher, J.O.; Johnson, R.K.; Lindquist, C.; Birch, L.L.; Goran, M.I. Influence of body composition on the accuracy of reported energy intake in children. *Obes Res* **2000**, *8*, 597-603.
49. Fontvieille, A.M.; Harper, I.T.; Ferraro, R.T.; Spraul, M.; Ravussin, E. Daily energy expenditure by five-year-old children, measured by doubly labeled water. *Journal of Pediatrics* **1993**, *123*, 200-207.
50. Franks, P.W.; Ravussin, E.; Hanson, R.L.; Harper, I.T.; Allison, D.B.; Knowler, W.C.; Tataranni, P.A.; Salbe, A.D. Habitual physical activity in children: the role of genes and the environment. *Am J Clin Nutr* **2005**, *82*, 901-908.
51. Gibson, A.R.; Ojiambo, R.; Konstabel, K.; Lieberman, D.E.; Reilly, J.J.; Speakman, J.R.; Pitsiladis, Y.P. Aerobic Capacity, Activity Levels and Daily Energy Expenditure in Male and Female Adolescents of the Kenyan Nandi Sub-Group. *PLoS ONE* **2013**, *8*, doi:http://dx.doi.org/10.1371/journal.pone.0066552.
52. Gondolf, U.H.; Tetens, I.; Hills, A.P.; Michaelsen, K.F.; Trolle, E. Validation of a pre-coded food record for infants and young children. *Eur J Clin Nutr* **2012**, *66*, 91-96, doi:http://dx.doi.org/10.1038/ejcn.2011.133.
53. Goran, M.I.; Hunter, G.; Nagy, T.R.; Johnson, R. Physical activity related energy expenditure and fat mass in young children. *Int J Obes Relat Metab Disord* **1997**, *21*, 171-178.
54. Goran, M.I.; Carpenter, W.H.; Poehlman, E.T. Total energy expenditure in 4- to 6-yr-old children. *Am J Physiol* **1993**, *264*, E706-711.
55. Goran, M.I.; Gover, B.A.; Nagy, T.R.; Johnson, R.K. Developmental changes in energy expenditure and physical activity in children: Evidence for a decline in physical activity in girls before puberty. *Pediatrics* **1998**, *101*, 887-891.
56. Goran, M.I.; Kaskoun, M.; Johnson, R.; Martinez, C.; Kelly, B.; Hood, V. Energy expenditure and body fat distribution in Mohawk children. *Pediatrics* **1995**, *95*, 89-95.
57. Gregory, J.W.; Greene, S.A.; Thompson, J.; Scrimgeour, C.M.; Rennie, M.J. Effects of oral testosterone undecanoate on growth, body composition, strength and energy expenditure of adolescent boys. *Clin Endocrinol (Oxf)* **1992**, *37*, 207-213.
58. Hallal, P.C.; Reichert, F.F.; Clark, V.L.; Cordeira, K.L.; Menezes, A.M.; Eaton, S.; Ekelund, U.; Wells, J.C. Energy expenditure compared to physical activity measured by accelerometry and self-report in adolescents: a validation study. *PLoS ONE* **2013**, *8*, e77036, doi:http://dx.doi.org/10.1371/journal.pone.0077036.

59. Henriksson, H.; Forsum, E.; Lof, M. Evaluation of Actiheart and a 7 d activity diary for estimating free-living total and activity energy expenditure using criterion methods in 15- and 3-year-old children. *Br J Nutr* **2014**, *111*, 1830-1840, doi:http://dx.doi.org/10.1017/S0007114513004406.
60. Henriksson, H.; Bonn, S.E.; Bergstrom, A.; Balter, K.; Balter, O.; Delisle, C.; Forsum, E.; Lof, M. A New Mobile Phone-Based Tool for Assessing Energy and Certain Food Intakes in Young Children: A Validation Study. *JMIR mHealth uHealth* **2015**, *3*, 8, doi:10.2196/mhealth.3670.
61. Hoffman, D.J.; Sawaya, A.L.; Coward, W.A.; Wright, A.; Martins, P.A.; de Nascimento, C.; Tucker, K.L.; Roberts, S.B. Energy expenditure of stunted and nonstunted boys and girls living in the shantytowns of Sao Paulo, Brazil. *Am J Clin Nutr* **2000**, *72*, 1025-1031.
62. Hoos, M.B.; Plasqui, G.; Gerver, W.J.; Westerterp, K.R. Physical activity level measured by doubly labeled water and accelerometry in children. *Eur J Appl Physiol* **2003**, *89*, 624-626.
63. Horswill, C.A.; Kien, C.L.; Zipf, W.B. Energy expenditure in adolescents during low intensity, leisure activities. *Med Sci Sports Exerc* **1995**, *27*, 1311-1314.
64. Hwang, J.; Lee, I.M.; Fernandez, A.M.; Hillman, C.H.; Lu, A.S. Exploring Energy Expenditure and Body Movement of Exergaming in Children of Different Weight Status. *Pediatr Exerc Sci* **2019**, *31*, 438-447, doi:10.1123/pes.2019-0006.
65. Ishikawa-Takata, K.; Kaneko, K.; Koizumi, K.; Ito, C. Comparison of physical activity energy expenditure in Japanese adolescents assessed by EW4800P triaxial accelerometry and the doubly labelled water method. *Br J Nutr* **2013**, *110*, 1347-1355, doi:http://dx.doi.org/10.1017/S0007114513000603.
66. Jhang, W.K.; Park, S.J. Energy Expenditure in Mechanically Ventilated Korean Children: Single-Center Evaluation of a New Estimation Equation. *Pediatric critical care medicine : a journal of the Society of Critical Care Medicine and the World Federation of Pediatric Intensive and Critical Care Societies* **2020**, *21*, e522-e529, doi:10.1097/pcc.0000000000002335.
67. Jiang, Z.; Yan, Q.; Su, Y.; Acheson, K.J.; Thelin, A.; Piguet-Welsch, C.; Ritz, P.; Ho, Z.C. Energy expenditure of Chinese infants in Guangdong Province, south China, determined with use of the doubly labeled water method. *Am J Clin Nutr* **1998**, *67*, 1256-1264.
68. Jindal, I.; Puyau, M.; Adolph, A.; Butte, N.; Musaad, S.; Bacha, F. The relationship of sleep duration and quality to energy expenditure and physical activity in children. *Pediatr Obes* **2021**, *16*, e12751, doi:10.1111/ijpo.12751.
69. Johnson, R.K.; Driscoll, P.; Goran, M.I. Comparison of multiple-pass 24-hour recall estimates of energy intake with total energy expenditure determined by the doubly labeled water method in young children. *J Am Diet Assoc* **1996**, *96*, 1140-1144.
70. Johnson, R.K.; Russ, J.; Goran, M.I. Physical activity related energy expenditure in children by doubly labeled water as compared with the Caltrac accelerometer. *Int J Obes Relat Metab Disord* **1998**, *22*, 1046-1052.
71. Joosten, K.F.; Verhoeven, J.J.; Hop, W.C.; Hazelzet, J.A. Indirect calorimetry in mechanically ventilated infants and children: accuracy of total daily energy expenditure with 2 hour measurements. *Clinical nutrition* **1999**, *18*, 149-152.
72. Kaskoun, M.C.; Johnson, R.K.; Goran, M.I. Comparison of energy intake by semiquantitative food-frequency questionnaire with total energy expenditure by the doubly labeled water method in young children. *Am J Clin Nutr* **1994**, *60*, 43-47.
73. Kim, E.K.; Ndahimana, D.; Ishikawa-Takata, K.; Lee, S.; Kim, H.; Lim, K.; Lee, I.S.; Tanaka, S.; Kim, Y.J.; Choi, Y.J.; et al. Validation of Dietary Reference Intakes for predicting energy requirements in elementary school-age children. *Nutr Res Pract* **2018**, *12*, 336-341, doi:10.4162/nrp.2018.12.4.336.
74. Komura, K.; Nakae, S.; Hirakawa, K.; Ebine, N.; Suzuki, K.; Ozawa, H.; Yamada, Y.; Kimura, M.; Ishii, K. Total energy expenditure of 10- to 12-year-old Japanese children measured using the doubly labeled water method. *Nutrition and Metabolism* **2017**, *14 (1) (no pagination)*, doi:http://dx.doi.org/10.1186/s12986-017-0226-y.
75. Krishnaveni, G.V.; Veena, S.R.; Kuriyan, R.; Kishore, R.P.; Wills, A.K.; Nalinakshi, M.; Kehoe, S.; Fall, C.H.; Kurpad, A.V. Relationship between physical activity measured using accelerometers and energy expenditure measured using doubly labelled water in Indian children. *Eur J Clin Nutr* **2009**, *63*, 1313-1319, doi:http://dx.doi.org/10.1038/ejcn.2009.95.
76. Lanigan, J.A.; Wells, J.C.; Lawson, M.S.; Lucas, A. Validation of food diary method for assessment of dietary energy and macronutrient intake in infants and children aged 6-24 months. *Eur J Clin Nutr* **2001**, *55*, 124-129.
77. Lee, J.M.; Saint-Maurice, P.F.; Kim, Y.; Gaesser, G.A.; Welk, G. Activity Energy Expenditure in Youth: Sex, Age, and Body Size Patterns. *J Phys Act Health* **2016**, *13*, S62-S70, doi:http://dx.doi.org/10.1123/jpah.2016-0014.
78. Lindquist, C.H.; Cummings, T.; Goran, M.I. Use of tape-recorded food records in assessing children's dietary intake. *Obes Res* **2000**, *8*, 2-11.

79. Livingstone, M.B.; Prentice, A.M.; Coward, W.A.; Strain, J.J.; Black, A.E.; Davies, P.S.; Stewart, C.M.; McKenna, P.G.; Whitehead, R.G. Validation of estimates of energy intake by weighed dietary record and diet history in children and adolescents. *Am J Clin Nutr* **1992**, *56*, 29-35.
80. Lopez-Alarcon, M.; Merrifield, J.; Fields, D.A.; Hilario-Hailey, T.; Franklin, F.A.; Shewchuk, R.M.; Oster, R.A.; Gower, B.A. Ability of the actiwatch accelerometer to predict free-living energy expenditure in young children. *Obes Res* **2004**, *12*, 1859-1865.
81. Lucas, A.; Ewing, G.; Roberts, S.B.; Coward, W.A. How much energy does the breast fed infant consume and expand? *British medical journal* **1987**, *295*, 75-77.
82. Maffeis, C.; Schutz, Y.; Chini, L.; Grezzani, A.; Piccoli, R.; Tato, L. Effects of dinner composition on postprandial macronutrient oxidation in prepubertal girls. *Obes Res* **2004**, *12*, 1128-1135.
83. Maffeis, C.; Schutz, Y.; Grezzani, A.; Provera, S.; Piacentini, G.; Tato, L. Meal-induced thermogenesis and obesity: is a fat meal a risk factor for fat gain in children? *J Clin Endocrinol Metab* **2001**, *86*, 214-219.
84. Maffeis, C.; Pinelli, L.; Zaffanello, M.; Schena, F.; Iacumin, P.; Schutz, Y. Daily energy expenditure in free-living conditions in obese and non-obese children: comparison of doubly labelled water (2H2(18)O) method and heart-rate monitoring. *Int J Obes Relat Metab Disord* **1995**, *19*, 671-677.
85. McGloin, A.F.; Livingstone, M.B.; Greene, L.C.; Webb, S.E.; Gibson, J.M.; Jebb, S.A.; Cole, T.J.; Coward, W.A.; Wright, A.; Prentice, A.M. Energy and fat intake in obese and lean children at varying risk of obesity. *Int J Obes Relat Metab Disord* **2002**, *26*, 200-207.
86. Montgomery, C.; Reilly, J.J.; Jackson, D.M.; Kelly, L.A.; Slater, C.; Paton, J.Y.; Grant, S. Validation of energy intake by 24-hour multiple pass recall: comparison with total energy expenditure in children aged 5-7 years. *Br J Nutr* **2005**, *93*, 671-676.
87. Nielsen, S.B.; Wells, J.C.; Fewtrell, M.S.; Eaton, S.; Grinham, J.; Reilly, J.J. Validation of energy requirement references for exclusively breast-fed infants. *Br J Nutr* **2013**, *109*, 2036-2043, doi:http://dx.doi.org/10.1017/S000711451200428X.
88. Nicklas, T.; Saab, R.; Islam, N.G.; Wong, W.; Butte, N.; Schulin, R.; Liu, Y.; Apolzan, J.W.; Myers, C.A.; Martin, C.K. Validity of the Remote Food Photography Method Against Doubly Labeled Water Among Minority Preschoolers. *Obesity (Silver Spring)* **2017**, *25*, 1633-1638, doi:10.1002/oby.21931.
89. O'Connor, J.; Ball, E.J.; Steinbeck, K.S.; Davies, P.S.; Wishart, C.; Gaskin, K.J.; Baur, L.A. Comparison of total energy expenditure and energy intake in children aged 6-9 y. *Am J Clin Nutr* **2001**, *74*, 643-649.
90. Ojiambo, R.; Konstabel, K.; Veidebaum, T.; Reilly, J.; Verbestel, V.; Huybrechts, I.; Sioen, I.; Casajus, J.A.; Moreno, L.A.; Vicente-Rodriguez, G.; et al. Validity of hip-mounted uniaxial accelerometry with heart-rate monitoring vs. triaxial accelerometry in the assessment of free-living energy expenditure in young children: The IDEFICS Validation Study. *Journal of applied physiology* **2012**, *113*, 1530-1536, doi:http://dx.doi.org/10.1152/japplphysiol.01290.2011.
91. Ojiambo, R.; Gibson, A.R.; Konstabel, K.; Lieberman, D.E.; Speakman, J.R.; Reilly, J.J.; Pitsiladis, Y.P. Free-living physical activity and energy expenditure of rural children and adolescents in the Nandi region of Kenya. *Ann Hum Biol* **2013**, *40*, 318-323, doi:http://dx.doi.org/10.3109/03014460.2013.775344.
92. Park, J.; Ishikawa-Takata, K.; Lee, S.; Kim, E.; Lim, K.; Kim, H.; Lee, I.S.; Tanaka, S. Comparison of daily physical activity parameters using objective methods between overweight and normal-weight children. *Journal of Sport and Health Science.* **2017**, *22*, doi:http://dx.doi.org/10.1016/j.jshs.2017.01.008.
93. Park, J.H.; Ishikawa-Takata, K.; Lee, S.J.; Kim, E.K.; Lim, K.W.; Kim, H.R.; Lee, I.S.; Tanaka, S. Association between daily step counts and physical activity level among Korean elementary schoolchildren. *Journal of Exercise Nutrition & Biochemistry* **2016**, *20*, 51-55, doi:https://dx.doi.org/10.20463/jenb.2016.09.20.3.8.
94. Perks, S.M.; Roemmich, J.N.; Sandow-Pajewski, M.; Clark, P.A.; Thomas, E.; Weltman, A.; Patrie, J.; Rogol, A.D. Alterations in growth and body composition during puberty. IV. Energy intake estimated by the youth-adolescent food-frequency questionnaire: validation by the doubly labeled water method. *Am J Clin Nutr* **2000**, *72*, 1455-1460.
95. Pfeiffer, K.A.; Schmitz, K.H.; McMurray, R.G.; Treuth, M.S.; Murray, D.M.; Pate, R.R. Physical activities in adolescent girls: variability in energy expenditure. *Am J Prev Med* **2006**, *31*, 328-331.
96. Prelack, K.; Yu, Y.M.; Dylewski, M.; Lydon, M.; Keaney, T.J.; Sheridan, R.L. Measures of Total Energy Expenditure and Its Components Using the Doubly Labeled Water Method in Rehabilitating Burn Children. *Jpen: Journal of Parenteral & Enteral Nutrition* **2017**, *41*, 470-480, doi:https://dx.doi.org/10.1177/0148607115597665.
97. Ramirez-Marrero, F.A.; Smith, B.A.; Sherman, W.M.; Kirby, T.E. Comparison of methods to estimate physical activity and energy expenditure in African American children. *Int J Sports Med* **2005**, *26*, 363-371.
98. Ramuth, H.; Schutz, Y.; Calonne, J.; Joonas, N.; Dulloo, A.G. Total energy expenditure assessed by doubly labeled water technique and estimates of physical activity in Mauritian children: analysis by gender and ethnicity. *Eur J Clin Nutr* **2020**, *74*, 445-453, doi:10.1038/s41430-019-0477-y.
99. Reilly, J.J.; Kelly, L.A.; Montgomery, C.; Jackson, D.M.; Slater, C.; Grant, S.; Paton, J.Y. Validation of Actigraph accelerometer estimates of total energy expenditure in young children. *Int J Pediatr Obes* **2006**, *1*, 161-167.

100. Reilly, J.J.; Montgomery, C.; Jackson, D.; MacRitchie, J.; Armstrong, J. Energy intake by multiple pass 24 h recall and total energy expenditure: a comparison in a representative sample of 3-4-year-olds. *Br J Nutr* **2001**, *86*, 601-605.
101. Rennie, K.L.; Livingstone, M.B.; Wells, J.C.; McGloin, A.; Coward, W.A.; Prentice, A.M.; Jebb, S.A. Association of physical activity with body-composition indexes in children aged 6-8 y at varied risk of obesity. *Am J Clin Nutr* **2005**, *82*, 13-20.
102. Roemmich, J.N.; Clark, P.A.; Berr, S.S.; Mai, V.; Mantzoros, C.S.; Flier, J.S.; Weltman, A.; Rogol, A.D. Gender differences in leptin levels during puberty are related to the subcutaneous fat depot and sex steroids. *Am J Physiol* **1998**, *275*, E543-551.
103. Roemmich, J.N.; Clark, P.A.; Walter, K.; Patrie, J.; Weltman, A.; Rogol, A.D. Pubertal alterations in growth and body composition. V. Energy expenditure, adiposity, and fat distribution. *Am J Physiol Endocrinol Metab* **2000**, *279*, E1426-1436.
104. Rush, E.C.; Plank, L.D.; Davies, P.S.; Watson, P.; Wall, C.R. Body composition and physical activity in New Zealand Maori, Pacific and European children aged 5-14 years. *Br J Nutr* **2003**, *90*, 1133-1139.
105. Salas-Salvado, J.; Barenys-Manent, M.; Recasens Gracia, M.A.; Marti-Henneberg, C. Influence of adiposity on the thermic effect of food and exercise in lean and obese adolescents. *Int J Obes Relat Metab Disord* **1993**, *17*, 717-722.
106. Salazar, G.; Vasquez, F.; Rodriguez, M.P.; Andrade, A.M.; Anziani, M.A.; Vio, F.; Coward, W. Energy expenditure and intake comparisons in Chilean children 4-5 years attending day-care centres. *Nutr Hosp* **2015**, *32*, 1067-1074, doi:10.3305/nh.2015.32.3.9263.
107. Salbe, A.D.; Nicolson, M.; Ravussin, E. Total energy expenditure and the level of physical activity correlate with plasma leptin concentrations in five-year-old children. *J Clin Invest* **1997**, *99*, 592-595.
108. Salbe, A.D.; Weyer, C.; Harper, I.; Lindsay, R.S.; Ravussin, E.; Antonio Tataranni, P. Assessing risk factors for obesity between childhood and adolescence: II. Energy metabolism and physical activity. *Pediatrics* **2002**, *110*, 307-314, doi:http://dx.doi.org/10.1542/peds.110.2.307.
109. Sijtsma, A.; Schierbeek, H.; Goris, A.H.; Joosten, K.F.; van Kessel, I.; Corpeleijn, E.; Sauer, P.J. Validation of the TracmorD triaxial accelerometer to assess physical activity in preschool children. *Obesity (Silver Spring)* **2013**, *21*, 1877-1883, doi:http://dx.doi.org/10.1002/oby.20401.
110. Singh, R.; Martin, B.R.; Hickey, Y.; Teegarden, D.; Campbell, W.W.; Craig, B.A.; Schoeller, D.A.; Kerr, D.A.; Weaver, C.M. Comparison of self-reported, measured, metabolizable energy intake with total energy expenditure in overweight teens. *Am J Clin Nutr* **2009**, *89*, 1744-1750, doi:http://dx.doi.org/10.3945/ajcn.2008.26752.
111. Sjoberg, A.; Slinde, F.; Arvidsson, D.; Ellegard, L.; Gramatkovski, E.; Hallberg, L.; Hulthen, L. Energy intake in Swedish adolescents: validation of diet history with doubly labelled water. *Eur J Clin Nutr* **2003**, *57*, 1643-1652.
112. Slinde, F.; Arvidsson, D.; Sjoberg, A.; Rossander-Hulthen, L. Minnesota leisure time activity questionnaire and doubly labeled water in adolescents. *Med Sci Sports Exerc* **2003**, *35*, 1923-1928.
113. Spadano, J.L.; Must, A.; Bandini, L.G.; Dallal, G.E.; Dietz, W.H. Energy cost of physical activities in 12-y-old girls: MET values and the influence of body weight. *Int J Obes Relat Metab Disord* **2003**, *27*, 1528-1533.
114. Sun, M.; Gower, B.A.; Nagy, T.R.; Trowbridge, C.A.; Dezenberg, C.; Goran, M.I. Total, resting, and activity-related energy expenditures are similar in Caucasian and African-American children. *Am J Physiol* **1998**, *274*, E232-237.
115. Tennefors, C.; Coward, W.A.; Hernell, O.; Wright, A.; Forsum, E. Total energy expenditure and physical activity level in healthy young Swedish children 9 or 14 months of age. *Eur J Clin Nutr* **2003**, *57*, 647-653.
116. Treuth, M.S.; Figueroa-Colon, R.; Hunter, G.R.; Weinsier, R.L.; Butte, N.F.; Goran, M.I. Energy expenditure and physical fitness in overweight vs non-overweight prepubertal girls. *Int J Obes Relat Metab Disord* **1998**, *22*, 440-447.
117. Urlacher, S.S.; Snodgrass, J.J.; Dugas, L.R.; Sugiyama, L.S.; Liebert, M.A.; Joyce, C.J.; Pontzer, H. Constraint and trade-offs regulate energy expenditure during childhood. *Sci Adv* **2019**, *5*, eaax1065, doi:10.1126/sciadv.aax1065.
118. Urlacher, S.S.; Snodgrass, J.J.; Dugas, L.R.; Madimenos, F.C.; Sugiyama, L.S.; Liebert, M.A.; Joyce, C.J.; Terán, E.; Pontzer, H. Childhood Daily Energy Expenditure Does Not Decrease with Market Integration and Is Not Related to Adiposity in Amazonia. *J Nutr* **2021**, *151*, 695-704, doi:10.1093/jn/nxaa361.
119. Valencia, M.E.; McNeill, G.; Haggarty, P.; Moya, S.Y.; Pinelli, A.; Quihui, L.; Davalos, R. Energetic consequences of mild Giardia intestinalis infestation in Mexican children. *Am J Clin Nutr* **1995**, *61*, 860-865.
120. van der Kuip, M.; de Meer, K.; Westerterp, K.R.; Gemke, R.J. Physical activity as a determinant of total energy expenditure in critically ill children. *Clinical nutrition* **2007**, *26*, 744-751.
121. Vasquez, F.; Salazar, G.; Andrade, M.; Vasquez, L.; Diaz, E. Energy balance and physical activity in obese children attending day-care centres. *Eur J Clin Nutr* **2006**, *60*, 1115-1121.
122. Vazquez Martinez, J.L.; Martinez-Romillo, P.D.; Diez Sebastian, J.; Ruza Tarrio, F. Predicted versus measured energy expenditure by continuous, online indirect calorimetry in ventilated, critically ill children

during the early postinjury period. *Pediatric critical care medicine : a journal of the Society of Critical Care Medicine and the World Federation of Pediatric Intensive and Critical Care Societies* **2004**, *5*, 19-27, doi:10.1097/01.PCC.0000102224.98095.0A.

123. Waling, M.U.; Larsson, C.L. Energy intake of Swedish overweight and obese children is underestimated using a diet history interview. *J Nutr* **2009**, *139*, 522-527, doi:http://dx.doi.org/10.3945/jn.108.101311.
124. Wells, J.C.; Cole, T.J.; Davies, P.S. Total energy expenditure and body composition in early infancy. *Archives of disease in childhood* **1996**, *75*, 423-426.
125. Wells, J.C.K.; Davies, P.S.W. Energy cost of physical activity in twelve week old infants. *Am J Human Biol* **1995**, *7*, 85-92, doi:https://dx.doi.org/10.1002/ajhb.1310070111.
126. Wells, J.C.; Davies, P.S. Estimation of the energy cost of physical activity in infancy. *Archives of disease in childhood* **1998**, *78*, 131-136.
127. Wells, J.C.; Hinds, A.; Davies, P.S. Free-living energy expenditure and behaviour in late infancy. *Archives of disease in childhood* **1997**, *76*, 490-494.
128. White, M.S.; Shepherd, R.W.; McEniery, J.A. Energy expenditure measurements in ventilated critically ill children: within- and between-day variability. *Jpen: Journal of Parenteral & Enteral Nutrition* **1999**, *23*, 300-304.
129. Wong, W.W. Energy expenditure of female adolescents. *J Am Coll Nutr* **1994**, *13*, 332-337.
130. Wren, R.E.; Blume, H.; Mazariegos, M.; Solomons, N.; Alvarez, J.O.; Goran, M.I. Body composition, resting metabolic rate, and energy requirements of short- and normal-stature, low-income Guatemalan children. *Am J Clin Nutr* **1997**, *66*, 406-412.
131. Yamada, Y.; Sagayama, H.; Itoi, A.; Nishimura, M.; Fujisawa, K.; Higaki, Y.; Kimura, M.; Aoki, Y. Total Energy Expenditure, Body Composition, Physical Activity, and Step Count in Japanese Preschool Children: A Study Based on Doubly Labeled Water. *Nutrients* **2020**, *12*, doi:10.3390/nu12051223.
132. Yamada, Y.; Sagayama, H.; Yasukata, J.; Uchizawa, A.; Itoi, A.; Yoshida, T.; Watanabe, D.; Hashii-Arishima, Y.; Mitsuishi, H.; Nishimura, M.; et al. Association between Water and Energy Requirements with Physical Activity and Fat-Free Mass in Preschool Children in Japan. *Nutrients* **2021**, *13*, doi:10.3390/nu13114169.
133. Zinkel, S.R.; Moe, M., 3rd; Stern, E.A.; Hubbard, V.S.; Yanovski, S.Z.; Yanovski, J.A.; Schoeller, D.A. Comparison of total energy expenditure between school and summer months. *Pediatr Obes* **2013**, *8*, 404-410, doi:http://dx.doi.org/10.1111/j.2047-6310.2012.00120.x.
